# A three-sided story: a biosystematic revision of genus *Datura* reveals novel tropane alkaloids for the first-time in certain species

**DOI:** 10.3389/fpls.2025.1555237

**Published:** 2025-05-02

**Authors:** Abeer Al-Andal, Mohamed Ewas, Abd El Raheim M. Donia, Asmaa M. Radwan, Mohamed N. S. Suliman, Elsayed Nishawy, Ahmed El-Shabasy, Eman Khames

**Affiliations:** ^1^ Department of Biology, College of Science, King Khalid University, Abha, Saudi Arabia; ^2^ Laboratory of Genomics and Genome Editing, Genetic and Cytology Unit, Plant Genetic Resources Department, Desert Research Center, Cairo, Egypt; ^3^ Department of Biochemistry, Faculty of Pharmacy, Tanta University, Tanta, Gharbia, Egypt; ^4^ Natural Products Unit, Medicinal and Aromatic Plants Department, Desert Research Center, Cairo, Egypt; ^5^ Botany and Microbiology Department, Faculty of Science, Girls Branch, Al-Azhar University, Cairo, Egypt; ^6^ Department of Biology, College of Science, Jazan University, Jazan, Saudi Arabia

**Keywords:** *Datura*, biosystematic revision, cytogenetics, molecular markers (CDDP, SCoT, ISSR), tropane alkaloids, gene expression

## Abstract

**Introduction:**

Datura, long considered an medicinal plant, represents a prospective source for discovering novel drugs for modern medicine. The Egyptian flora encompasses six Datura genotypes, including D. innoxia, D. ferox, D. metel and three forms of D. stramonium (stramonium, tatula and inermis). However, the taxonomic status of Datura genus remains controversial.

**Methods:**

Our study aims to clarify the phylogenetic relationships among Egyptian Datura via contemporary molecular taxonomy techniques, including ISSR, SCoT, CDDP, as well as cytogenetics assessment, and chemical taxonomy, using total- and tropane-alkaloid and UV spectroscopic pattern.

**Results:**

Based on our results, the three forms of D. stramonium (stramonium, tatula and inermis) are closely related taxa, although there were some significant differences, suggesting the need to upgrade inermis to level of subspecies (Datura stramonium subsp. Inermis). The GC-MS results identified 31 tropane alkaloids. Out of which, seven were described in a qualitative manner in genus Datura, which enhances these genotypes’ medical and economic value. Expression level of the genes PMT, TR1, TR2, H6H, HDH and AT4 influenced the tropane alkaloids accumulation within the genotypes examined.

**Discussion:**

To date, this is the first study to identify the evolutionary relationship of the genus Datura combining molecular and chemical taxonomy, and to quantify the TAs and the genes involved in their biosynthesis among Datura genotypes.

**Conclusion:**

This study is significant since drug development strategies and enhanced therapeutic usage of Datura heavily depend on comprehensive knowledge of the species and subspecies’ molecular and phytochemical variability.

## Graphical Abstract

Graphical abstract on the biosystematic revision of Egyptian *Datura* genotypes including phytochemical investigation based on total phenolic, flavonoids, alkaloids and tropane alkaloids alongside with identification of plant material using molecular markers, cytogenetic and gene expression analysis and finally, evaluation of genetic variability via Principal Components Analysis (PCA) and Hierarchical Cluster Analysis (HCA).

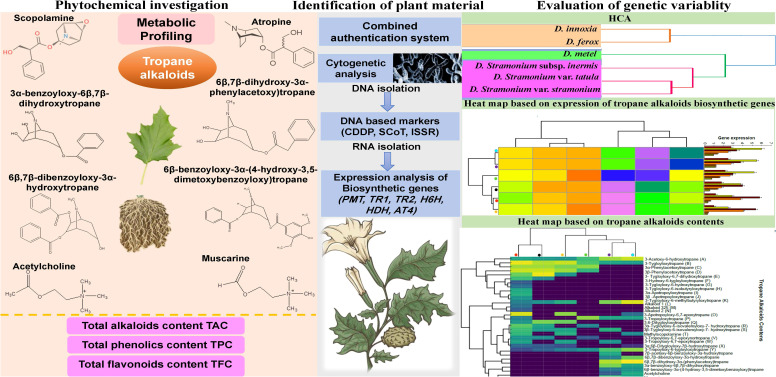

## Highlights

- Phytochemical profiling of investigated *Datura* genotypes was carried out.
- Integrated authentication using phytochemical and molecular profiles is essential.
- Genetic identifiers consisted of molecular markers, Chromosome counting and karyotyping.
- Multivariate analysis identified valid metabolites for genotype grouping.
- The phenolic spectra are mostly governed by inheritance.

## 1 Introduction

Since ancient times, medicinal plants have been employed in healing (Sofowora et al., 2013). Medical plants have begun to be considered as an essential source in both curing and avoiding a variety of diseases (Rakotoarivelo et al., 2015). In many regions of the world, where about 80% of the population only uses traditional medical treatments, medicinal plants continue to play a vital role in human healthcare systems. They also contribute significantly to biodiversity worldwide (Asigbaase et al., 2023). Plant-based products constitute the primary source of healthcare for more than four billion people in underdeveloped countries (Ekor, 2014). In developed nations, the uses of alternative remedies and herbal medicines have grown dramatically during the last 20 years (Ekor, 2014). Medicinal plants are used as the raw material by pharmaceutical companies to create other semi-synthetic pharmacologically active ingredients that are included in many medications (Hao and Xiao, 2020). Approximately 30% of medications sold globally include substances originating from plants (Asigbaase et al., 2023).

Egypt is the meeting point for floristic aspects from four phytogeographical regions: African Sudano-Zambesian, Asian Irano-Turanian, Afro-Asiatic Saharo-Sindian, and Euro-Afro-Asiatic Mediterranean (El Hadidi and Hosni, 2020). Egypt is also the most arid country in Africa’s northern region, with desert ecosystems making up the majority of the country. Hot ecosystems are classified as either dry or hyper-arid regions. Hyper-arid regions are distinguished by hot summers (mean temperature of 30°C in the warmest month) and an average winter rainfall of 30 millimeters per year.

Egypt’s flora comprises a significant number of families and genera (120 families, 742 genera, and 2088 species), as well as numerous oligotypic families, depicted by only one or a handful of species (Khedr et al., 2002). The genus *Datura* Linnaeus (1753: 179) (*Datureae, Solanoideae, Solanaceae*) contains 12-14 species (Jiao et al., 2002), mostly annual or perennial plants (Hassan et al., 2019). A global weed, *Datura* grows in numerous disturbed habitats. It is a known plant that grows in both Egypt and the Kingdom of Saudi Arabia (Al-Shaikh and Sablay, 2005). In addition to its high toxicity, it is distinguished by its narcotic, psychedelic, and therapeutic qualities (Dupin and Smith, 2018; Hassan et al., 2019). Previously utilized in folk medicine (Hassan et al., 2019). The tribe *Datureae* first appeared in Andean areas before spreading to non-Andean countries and North America. The majority of *Datura* species are indigenous to tropical dry forests in Mexico, arid regions in Mexico’s north and the southwestern United States (**Supplementary Table 1**) (Dupin et al., 2017a; Dupin and Smith, 2018). There is a disagreement about how many species of the genus *Datura* exist in Egypt. Täckholm (1974) (Täckholm, 1974) described 5 *Datura* species from Egypt, including *D. stramonium* L., *D. tatula* L., *D. innoxia* Mill., *D. metel* L., and *D. fastuosa* L. Whereas Hepper (1998) (Hepper, 1998) and Boulos (2002, 2009) (Boulos, 2002; Boulos, 2009) identified just 3 species including *D. stramonium, D. innoxia*, and *D. metel*, he considered *D. tatula* and *D. fastuosa* to be synonyms of *D. stramonium* and *D. metel*, respectively. Recently, the species *Datura ferox* was recorded for the first time in St. Catherine by Rabei et al. (2019) (Rabei et al., 2019). The biodiversity of this genus is attributed to the variation in genetic and chemical characteristics (Ibrahim et al., 2018).

The morphology-based taxonomy is still largely unresolved because of 1) the extremely high number of nominal species that have been recorded, 2) the low number of morphological traits that can be used to identify species, 3) the elevated level of intraspecific morphological variations, and 4) the inability to distinguish certain species morphologically. Because of this, over half of the species are thought to be interchangeable or listed under wrong taxon (Hua et al., 2019). A number of molecular taxonomy for distinguishing between plant species and varieties, and to explore the evolutionary has history been employed (Ewas, 2023). However, the rapid progress of genomics research allows for the application of gene-targeted approach in addition to random DNA markers (Andersen and Lübberstedt, 2003). Conserved DNA-Derived Polymorphism (CDDP) markers have developed from widely known plant genes that respond to environmental stresses, as well as biological processes (Bidyananda et al., 2024). This technique culminates in the development of functional markers which are closely linked to certain plant phenotypes. Plant studies using CDDP markers have indicated that genetic diversity is regulated by geographical distances and the degree of gene exchange among plant groups and sexes under various environmental circumstances (Bilčíková et al., 2021; Haffar et al., 2022). These markers have been effectively employed to assess genetic diversity in several plant species, *in-situ* and *ex-situ* conservation attempts, and genotype identification (Liu et al., 2020). CDDP markers provide various benefits, particularly convenience, low cost, and an extensive range of polymorphisms that may be used to generate specific trait markers (Bidyananda et al., 2024).

Chemotaxonomy is a biological categorization system that uses similarities and differences in the structure of specific molecules between organisms to classify them. Over the past ten years, there has been an increasing interest in creating a more uniform chemical taxonomy and ontology. Though they have been used as systematic markers for the same duration as morphological features, secondary metabolites generated by organisms have remained subjective until recently. The argument is that proteins are more accurate markers of genetic links than physical traits because they are more tightly regulated by genes and less susceptible to natural selection. The most frequently investigated chemicals are peptides, proteins, amino acids, and nucleic acids (Waterman, 1998). At the chemical level, the plants of the genus *Datura* are arguably the most well-known—and notorious—plants in Earth’s history. Naturalized in all of the world’s temperate zones. Similar to other members of the *Solanaceae* family, *Datura* plants are abundant in bioactive phytochemicals. The alkaloids in *Datura* are what have solidified these plants’ place in the medicine, religion, history, and folklore of many civilizations worldwide. It is also worth mentioning that phenolics, flavonoids, steroids, amides, acylsugars and other substances within these plants have been identified and separated (Cinelli and Jones, 2021). *Datura* continues to be the focus of a very extensive research since several of the tropanes, indoles, pyrrolidines and other alkaloids found in the plant have both therapeutic and sneaky toxicity. Clarifying the unique and potent characteristics of these plants and the compounds that they give rise to, has been the focus of many multidisciplinary endeavors for more than a century, with investments from the fields of pharmaceutical development, plant breeding, analytical chemistry, genetic engineering, ethnobotany and biological evolution (Cinelli and Jones, 2021).

Alkaloids are among these plant metabolites; we specifically discuss tropane alkaloids (TA) here, with (-)-hyoscyamine and (-)-scopolamine (sometimes called hyoscine) being the most significant naturally occurring TAs. These alkaloids have been detected in high amounts, especially in *Datura ferox*, *stramonium*, and *innoxia*. TAs have a very different pattern within *Datura* species (Cinelli and Jones, 2021). Considering their anticholinergic effect, they are frequently utilized in medical treatments to be antispasmodics for alleviating tension within the smooth muscles of the digestive and urinary channels, as mydriatics in ophthalmoscopic examinations, and for avoiding nausea while traveling (Jaremicz et al., 2014). Pharmacological synthetic attempts to create such TAs have proven commercially impractical given its stereochemistry, consequently they are mostly derived from cultivated plants (Christen, 2000; Grynkiewicz and Gadzikowska, 2008).

The current study aims to clarify the importance of identifying phylogenetic relationships among specific *Datura* genotypes within Egyptian flora via contemporary molecular taxonomy techniques, such as ISSR, SCoT, CDDP, chromosome numbers and karyomorphological assessment. In addition, we are proposing a chemical taxonomy based on the total alkaloid and TA contents along with the UV spectroscopic patterns of total phenolic and flavonoids (**Graphical Abstract**).

## 2 Materials and methods

### 2.1 Plant material, geographical and ecological data

To assess the phenotypic, genetic and chemical variability of the examined forms, the current study was based on an investigation of *Datura* specimens housed in the Agricultural Museum (CAIM) and the National Gene Bank (NGB), National Research Centre (CAIRC), Cairo, Egypt and Department of Biology, College of Science, King Khalid University, Abha, Saudi Arabia. Seeds of these genotypes under study were gifted to our Research group after collecting from environmentally diverse geographical locations including the Red Sea, St. Catherine in South Sinai and the Nile Delta, then stored in the storage unites of NGB by Dr. Omran G. Ibrahim, Desert Research Center. The plant materials were identified by Dr. Omran G. Ibrahim, DRC and the voucher herbarium specimens were deposited in the herbarium of Desert Research Center (CAIH) with Code Number: CAIH-1027-S. Furthermore, specimens from the online virtual herbaria [the Royal Botanic Garden, Kew (K) herbarium, the Harvard University Herbaria & Libraries (HUH) (https://huh.harvard.edu/), the Botanic Garden and Botanical Museum Berlin (B) herbarium (https://www.bgbm.org/en), the New York Botanical Garden (NYBG) (https://www.nybg.org/), and the JSTOR Global Plants database (http://plants.jstor.org) were additionally examined. To eliminate ecological variation and prepare the six *Datura* genotypes for ensuing comparative morphological, molecular, and biochemical investigations, field experiments were carried out in the experimental field of Tanta University in Tanta, Egypt, during 2024. For every genotype, 18 populations were chosen. The taxonomic analysis of twenty-four morphological features (such as stem, leaf, inflorescence, fruit, and pollen features See **Supplementary Table 2**) was conducted using eight individuals from each population. For each genotype, eight fresh specimens were chosen for anatomical analysis under a light microscope (AmScope M158C-SP14-E 40X-1000X). The Desert Research Center Herbaria (DRCH) in Cairo, Egypt, is where voucher specimens of the taxa under study were placed.

### 2.2 Morphological examination

A light microscope (AmScope M158C-SP14-E 40X-1000X) was used to analyze the stem’s and leaf’s epidermal system. Eight longitudinal slices in acropetal sequence were used to examine the young fruit’s anatomy.

### 2.3 Data analysis

Binary code (0 or 1) was created from each of the morphological feature states. To analyze the similarity of binary info via SPSS (version 20.0, 2011), differences between the forms were assessed using a simple matching measure method, which uses a similarity matrix was produced based on the morphological results of the genotypes under study.

### 2.4 Scanning electron microscope and sample preparation

The floral buds of the *Datura* genotypes were used to harvest fresh anthers. A Joel 1200 EX II SEM was used to scan pollen samples at 20 kV after preparation. Ten randomly chosen grains from ten individuals per genotypes were averaged to determine size. The terminology for the pollen that was employed in this work was based on Punt et al. (1994) and Erdtman (1952) (Erdtman, 1952; Punt et al., 1994).

### 2.5 Chromosome counting

Ten plants from various populations had well grown root tips cut off. Each genotype was subsequently treated with colchicine (0.25%) for two hours and fixed in Carnoy solution (3 ethanol:1 acetic acid) for four hours at 25°C. In accordance with Fukui & Nakayama’s (1996) (Fukui and Nakayama, 1996) procedure, samples were then thoroughly cleaned with distilled water and soaked using an enzymatic mixture (4% cellulose, 1% pectinase, 75 Mm KCl, and 7.5 Mm EDTA) and fixed on a glass slide in a moisture chamber at 37°C for 40 minutes before being stained using 1% Aceto orcein (Lobal Chemie, Mumbai, India).

### 2.6 Karyotyping

Metaphase spread analysis was used to determine the karyotypes of root tips from each genotype under study. Cells in mitotic metaphase were subjected to chromosome counting using a light microscope (Leica DM 2500, Wetzlar, Germany). Eight easily visible and widely distributed metaphases of eight individuals from various populations were chosen for each genotype under study, and they were captured on camera utilizing excellent quality mechanized karyotyping software analyzing (Leica CW4000) and the Image Processing Analysis System Standard. The centromere location, which determines a karyotype, was taken into consideration when placing the metaphase chromosomes in a decreasing pattern of size. For every pair of chromosomes, the classification of Levan et al. (1964) (Levan et al., 1964) was applied. The average total length of every single chromosome (c) was computed by taking the mean lengths of the short arm (s) and long arm (l) (c=s+l). For every single pair of chromosomes, the mean relative length (RL) was determined using the formula (c/sum c) × 100. The centromere’s location was determined by calculating the mean centromeric index (ci) for each pair of chromosomes by (s/c) × 100. The chromosome was classified as acrocentric if the (ci) value was within 0 and 12.5; telocentric if it was zero; metacentric if it was within 37.5 and 50.0; submetacentric if it was within 25.0 and 37.5; and sub-telocentric if it was within 12.5 and 25.0. The Romero Zarco formula (1986) (Zarco, 1986) for intrachromosomal asymmetry (Equation 1) was used to quantify Karyotypeasymmetrical for the relationships amongst the chromosome arms ineach genotype under study. The variance in chromosome length was also estimated using the Romero Zarco indicator (1986) (Zarco, 1986) according to Pearson’s dispersion factor and the intrachromosomal asymmetry.

### 2.7 Samples preparation, molecular amplification and gene detection

DNA was extracted from *Datura* leaves and roots and analyzed at the DRC’s laboratories, and Tanta University (TU), Faculty of Pharmacy’s molecular biology labs in Tanta, Gharbia, Egypt. DNeasy plant Mini Kit (QIAGEN) was employed for bulked DNA extraction, as was a DNeasy-like approach (Alexander et al., 2007). The ISSR and SCoT markers were amplified by PCR employing a 25 ng DNA template and the temperature cyclic profile given below. One U Taq DNA polymerase (Promega^®^), 2.5 mM MgCl_2_, 10 pmol primer, 1X TBE buffer, 25 ng DNA template in an overall reaction volume of 25 μl, and 0.25 mM dNTPs were all included in the reaction mixture. A computerized thermal cycler model (Techne 512, Bibby ScientificTM, UK) was used to perform the DNA amplifications. It was set up for one denaturation cycle at 94°C for four minutes, followed by forty-five cycles of one min. at 94°C, one min. at 57°C, and two min. at 72°C for elongation. The primer extension segment was then prolonged to ten min., the amplified products were identified by electrophoresis using agarose (1.5% w/v in 1 TBE buffer) and ethidium bromide (0.5 g/ml). A 100 bp DNA ladder mix was used as a standard, and 15 μl of the amplified DNA product was injected to each well. Twelve primers were obtained from Metabion International AG in Germany; six of these were chosen for ISSR evaluation (**Supplementary Table 3**), and six more were chosen for SCoT analysis (**Supplementary Table 4**).

Regarding the CDDP system, six gene markers out of 20 (**Supplementary Table 5**) were polymorphic and amplified (Collard and Mackill, 2009) and therefore used for genotyping. An overview of the CDDP markers found in *Datura* genotypes is provided (**Supplementary Tables 5**, **11**). The reaction mix (25.0 μL) included 6.0 μL distilled water, 4.0 μL (10 μM) primer, 5.0 μL (80.0 ng) DNA template, and 10.0 μL master mix (AddBio, Korea). The 38 cycles of amplification were performed in a Thermo-cycler (Applied Biosystem) and included a denaturation phase at 94°C for one minute, an annealing phase at 50°C for CDDP primers for one minute, and an extension phase at 72°C for 2 minutes. There was also a final extension phase of nine minutes at 72°C and an initial denaturation phase of seven minutes at 94°C. A gel documentation system was used for observing the amplified products after they had been electrophoresed on a 1.7% agarose gel stained using Ethidium Bromide (0.6 g/mL).

### 2.8 Measurement of total phenolic

To determine the total phenolic content, *Datura* leaf samples (1g fw) that had been crushed finely in liquid nitrogen were suspended in 20 mL of methanol and swirled for three hours at 23°C in the dark, before centrifuged for 15 minutes at 12,000×g. As previously mentioned by Ewas et al. (2022) (Ewas et al., 2022), 2mL of saturated sodium carbonate solution (roughly 75 g L^−1^) was added to the reaction mix after the total phenolic content was ascertained via the Folin–Ciocalteau reagent. Following a two-hour incubation period at 25^°^C, the optical density was obtained at 765 nm. The results have been displayed as gallic acid equivalents (mg 100 g^−1^ fw), with gallic acid serving as the reference standard.

### 2.9 Measurement of total alkaloids

In accordance with John et al. (2014) (John et al., 2014), the total phenolic content was measured in a continuous extraction (soxhlet) device. To achieve this, 100g of leaves were ground and then extracted with methanol for 24 hours. After filtering the extract, the methanol was dried by vacuum-evaporating it at 45°C in a rotary evaporator. A portion of this residue was filtered after being dissolved in 2 N HCl. Ten milliliters of chloroform were used three times to wash one milliliter of this solution after it had been moved to a separatory funnel. 0.1 N NaOH was used to bring this solution’s pH down to neutral. This solution was then mixed with 5 milliliters of BCG solution and 5 milliliters of phosphate buffer. After vigorously shaking the mixture, the complex that developed was extracted using 1, 2, 3, and 4 milliliters of chloroform. Chloroform was used to dilute the extracts to desired volume after they were collected in a 10-ml volumetric flask.

### 2.10 Qualitative analysis via HPTLC for alkaloids

In accordance with Jaremicz et al. (2013) (Jaremicz et al., 2014), the leave samples (about 100 mg) that had been lyophilized (Lyovac GTA; Fin-Aqua-Sohlberg Co., Finland) and thoroughly crushed were sonicated (Sonorex Digitec DT512H; Bandelin, Germany; at 25°C for 3 x 15 minutes) in polypropylene test tubes using an extraction solvent consisting of chloroform:methanol:25% ammonia (15:15:1, v/v/v) (20 milliliters). Chloroform (10 mL) and 0.5 M H_2_SO_4_ (10 milliliters) were used to separate the remains after the solvents were filtered and evaporated at 40°C under lower pressure. Chloroform (3 x 20 milliliters) was used to extract the acidic layer after it had been made alkaline with 25% ammonia (pH = 9.0). After drying over anhydrous sodium sulfate, the combination of organic phases was filtered and evaporated at 40°C under lower pressure. Prior to analysis, the remains were kept at 4°C after being reconstituted in only one milliliter of methanol. Chloroform (3 x 20 mL) was used for extracting the liquid medium, which was the entire volume obtained after two months of cultivation and was approximately 15 milliliters. It was made alkaline (pH = 9.0) with 25% ammonia. Under reduced pressure (40°C), the gathered organic layers were dried over anhydrous sodium sulfate and then evaporated. After being reconstituted in one milliliter of methanol, the samples were kept at 4°C until analysis. Regarding chromatography, all samples and standards were separated on HPTLC plates (5 cm x 20 cm Si60 F254, Merck, Darmstadt, Germany) for qualitative analysis. A mobile phase made up of chloroform, methanol, acetone, and 25% ammonia (75:15:10:1.6) was used.

### 2.11 Alkaloid’s extraction

Alkaloids from leaves of all *Datura* samples were extracted using 50 mg of dry matter according to Amdoun et al., 2010 (Amdoun et al., 2010). After the roots were pulverized and dried for 48 hours at 40°C, 50 milligrams were extracted for five minutes using 6 milliliters of hexane. The hexane phase, that included fat compounds but not alkaloids, was thrown away. After centrifugation, 12 milliliters of HCl 0.1 N were added for 10 minutes, then NH_4_OH (28%) was used to get the pH reach to 10. Using the same amount of chloroform, the aqueous phase was purified and extracted three times. Anhydrous Na_2_SO_4_ was then used to dry the organic phase. The remainder was resuspended in 5 milliliters of dichloromethane, which had been filtered at a pore size of 0.2 µm, after the organic phase had been removed by evaporation.

### 2.12 GC/MS (screening of total alkaloids content) analysis

Following the procedures of Nguyen et al. (2015) (Nguyen et al., 2015), the filtrates were subjected to GC/MS analysis in order to test for TAs in a subset of *Datura* roots. According to Nguyen et al. (2015) (Nguyen et al., 2015) GC/MS analysis was carried out using a Trace GC Ultra instrument that was connected to a DSQ II mass spectrometer (Thermo Fischer Scientific, Waltham, USA) and had a Triplus autosampler. A low-bleed VF-5 MS column (30 m × 0.25 mm × 0.25 µm) (Varian Inc., Grenoble, France) was used to examine alkaloids. At 250°C, one microliter was administered in splitless mode. The oven’s temperature was maintained at 40°C for one minute, then raised by 30°C per minute to 130°C, then by 10°C per minute to 280°C, and finally maintained at 280°C for five minutes. One mL min−1 of helium was utilized as the carrier gas. The ion supply and transfer line had temperatures of 200°C and 300°C, respectively. Mass spectra were captured at 4.7 scans per second, with a scanning range of 30 to 600 m/z. The mass spectrum of each TA was compared to the NIST05 database and the laboratory’s own database, which was issued by Nguyen et al. (2015) (Nguyen et al., 2015).

### 2.13 UV spectroscopic assay

Six *Datura* genotype extracts were made employing methanol of high-performance liquid chromatography (HPLC) grade (Fisher Scientific^®^). The optimal amount of extract was ascertained through preliminary analysis. These experiments led to a fixed dosage of 0.1 mg dry leaves extract/ml. Utilizing a spectrophotometer, the absorbance of the extracts was determined at wavelengths between 250 and 400 nm, since the methanol spectra of flavones and flavonols display two noticeable absorption peaks in this range. These two peaks are called Band I (usually between 300 and 380 nm) and Band II (usually between 240 and 280 nm) (Mabry et al., 1970; Ewas, 2023). The studies were carried out twice.

### 2.14 Multivariate data analysis

Using the average linkage strategy based on the Euclidean distance across genotypes, unsupervised chemometric techniques like PCA and HCA were carried out using the Unscrambler X 10.4 CAMO program (Computer Aided Modelling, AS, Norway) in order to distinguish between various Datura genotypes.

### 2.15 Quantitative measurement of flavonoids

With minor adjustments, the flavonoid concentrations were measured using a colorimetric test technique of Shi et al. (2012) (Shi et al., 2012). In short, the calibration linear with function was established using rutin as a standard:


(1) $$\begin{array}{l} A\;=\;8.0045\;C+0.0914;\\ r\;=\;0.9959\;\;\left(r\;=\;linear\;range\right) \end{array}$$

Samples weighing 1.00 g were weighed, and flavonoids were extracted using a supersonic (KQ-300DE, Kunshan Ultrasonic Equipment Co., China) for 30 minutes using 10 mL of 60% ethanol aqueous. These specimens underwent a further centrifugation at 3000 g. After all of the supernatant was moved to a 25 mL volumetric flask, it was fixed in 25 mL of aqueous 60% ethanol. Pipetting 1.5 mL of each extract and 4.5 mL of distilled water into a 25 mL tube, 1 mL of 5% (w v^−1^) NaNO2 solutions were added. One milliliter of the 0% (w v^−1^) Al (NO3)3 solutions was added to the mixture following a 6-minute incubation period. After 6 minutes, 10 mL of 4% (w v^−1^) NaOH solutions were added, and the mixture was fixed to 25 mL with 60% ethanol aqueous. Following a 15-minute reaction, the mixture’s absorbance was measured at 510 nm using a spectrophotometer (SP-1901, Shanghai Spectrum Instruments Co., China) in comparison to a blank that contained 5 mL of extraction solvent. The total flavonoid content was calculated as mg rutin equivalent per g dry weight (DW) using the mean of three independent analyses of the samples conducted in duplicate.

### 2.16 Expression analyses of tropane alkaloid’s biosynthetic genes

As directed by the manufacturer, 200 U of Invitrogen’s M-MLV reverse transcriptase and 3 μg of total RNA extracted from the roots of Datura genotypes were used to create the first-strand complementary DNA (cDNA). Invitrogen’s Trizol solution was used to isolate total RNA. Each alkaloid’s biosynthesis gene was amplified in 31 cycles using RT-PCR using a 400 bp fragment using first-strand cDNA as a template. Additionally, the action was increased for 24 cycles as an internal control measure. Using an optical 96-well plate (Thermo Scientific) and an Applied Biosystems AB StepOnePlus PCR apparatus, RT-PCR was performed using the SYBR Premix Kit F-415. The relative gene expression was calculated using a relative quantification approach. **Supplementary Table 6** contains a list of every primer utilized in this inquiry.

## 3 Results

### 3.1 Morphological taxonomy

#### 3.1.1 Characteristic morphological features of the studied forms of genus *Datura*

**Figure 1:**
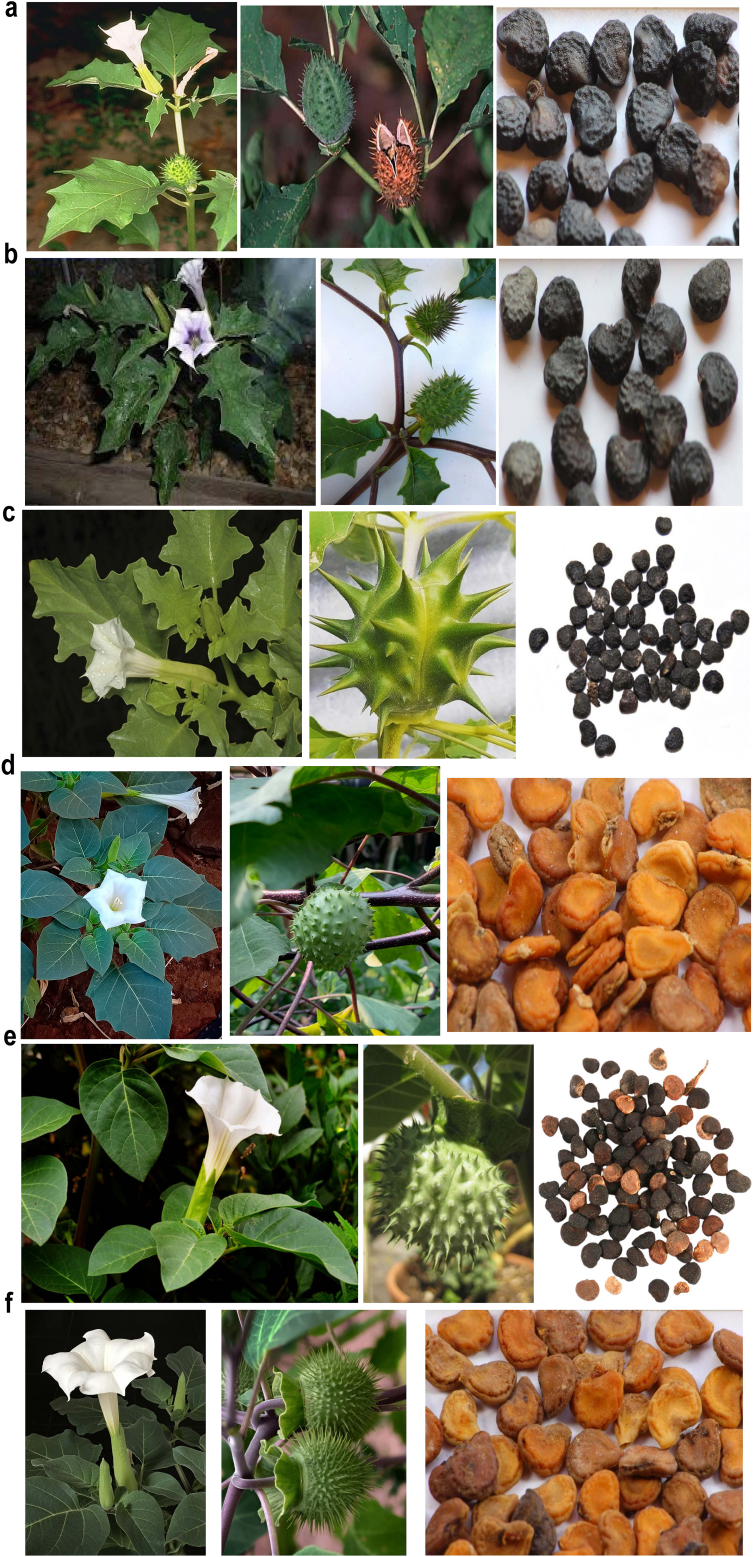
Morphological characterization for the leaves, stems, flowers, capsules and seeds of Egyptian *Datura* genotypes including, (**a**) *D. stramonium* form *stramonium* (**b**) *D. stramonium* form *tatula*. (**c**) *D. stramonium* subsp. *Inermis*. (**d**) *D. metel*. (**e**) *D. ferox*. (**f**) *D. innoxia*.

The morphological diversity observed across the six *Datura* species and varieties demonstrates both shared traits and significant differences that are important for their classification and ecological adaptation. Stem height varies significantly among the species, with *Datura innoxia* and *Datura metel* reach up to 1.5 meters, whereas, *Datura ferox* grows less than 1 meter. *D*atura *stramonium* form *stramonium* can reach up to 1 meter, other varieties of *Datura stramonium*, including var. *inermis* and var. *tatula* grow to less than 1 meter. This variation in height may reflect adaptation to different environmental conditions. The growth habit also differs: *D. stramonium form stramonium* and *D. ferox* have an erect growth form, while *D. innoxia* and *D. metel* display a spreading habit, suggesting they may occupy different ecological niches or use distinct growth strategies. The stem color varies across species: *D. stramonium form stramonium* and *D. inermis* are yellowish green, *D. stramonium form tatula* is purplish green, *D. innoxia* is greyish green, *D. metel* is dark green to purplish, while *D. ferox* is yellowish green (**Figure 1**; **Supplementary Table 2**). The leaf characteristics of the six studied *Datura* genotypes show notable similarities and differences, which can be compared as follows. *D. innoxia*, *D. metel*, and *D. ferox* possess the largest leaves, reaching lengths of 100 to 200 mm and widths of up to 120 mm. In contrast, *D. stramonium* form *stramonium*, form *tatula*, and form *inermis* feature smaller leaves, with *D. stramonium* form *stramonium* and form *inermis* measuring up to 150 mm in length and a width of up to

120 mm. The variety *D. stramonium form tatula* has leaves that are less than 150 mm in length and width, which point to differences between varieties within the same species. In terms of shape, *D. ferox* is characterized by its broadly ovate leaf shape, while *D. stramonium* form *stramonium*, form *inermis* and *D. metel* exhibit ovate-elliptic shapes, while *D. stramonium* form *tatula* and *D. innoxia* have elliptic-ovate leaves, showing a range of shapes across the species (**Figure 1**; **SupplementaryTable 2**). Hair density varies significantly among the species. *D. ferox* has dense hairs on both the abaxial and adaxial surfaces, providing it with a unique texture compared to the other species. In comparison, *D. innoxia* and *D. metel* have sparse hairs on both surfaces, indicating a smoother texture. For *D. stramonium* form *stramonium* and form *tatula* are sparsely hairy on the abaxial surface but have dense hairs on the adaxial surface. Notably, *D. stramonium form inermis* lacks hairs entirely, making it distinct in the group. Regarding trichome types, *D. ferox* has only eglandular trichomes. *D. innoxia* and *D. metel* also possess eglandular trichomes. In contrast, *D. stramonium* form *stramonium* and form *tatula* contain both eglandular and glandular trichomes. Notably, *D. stramonium form inermis* lacks trichomes entirely. This variation in trichome type may influence the plants’ defense mechanisms against herbivores and environmental stressors (**Supplementary Figures 2A-Y** and **Supplementary Table 2**). The flower characteristics of the six studied *Datura* species and varieties reveal notable differences and similarities that are important for their identification and ecological adaptations. *D. stramonium form stramonium* produces flowers with a corolla length of less than 100 mm. The corolla is typically white, and the calyx length is up to 40 mm with a width of 15 mm. The calyx is light green, and it features 5 or 6 teeth that can reach a length of up to 6 mm. *D. stramonium* form *tatula* has flowers with a distinct violet corolla that distinguishes it from other varieties. It reaches a length of up to 100 mm. The calyx length is less than 50 mm, with a width of 15 mm with has 5 calyx teeth that grow to a length of up to 8 mm. The *D. stramonium* form *inermis* has flowers with a corolla length of up to 100 mm. The corolla is typically white, and the calyx length is less than 40 mm, with a width of 15 mm. The calyx is light green, and it has 5 teeth that reach a length of up to 6 mm. On the other hand, *D. innoxia* produces larger flowers, with a corolla length ranging from 100 to 150 mm. The corolla is usually white, while the calyx length measures between 50 and 70 mm and the width are 15–20 mm. The calyx is yellowish green, and it can have 5 or 6 teeth, which can reach a length of up to 8 mm. *D. metel* has larger flowers, with a corolla length of 150 to 200 mm. The corolla color is typically white, but it may sometimes have purplish hues. The calyx length is also significant, measuring between 50 and 70 mm, with a width ranging from 20 to 25 mm. The calyx is light green and features 5 or 6 teeth that can be as long as 10 mm. *D. ferox* produces flowers with a corolla length of 100 to 150 mm, similar to some other varieties. The corolla is predominantly white. The calyx length is between 30 and 50 mm with a width of 10 to 15 mm. The calyx is light green, and it typically has 5 teeth that can reach a length of up to 6 mm (**Figure 1**; **Supplementary Table 2**). All forms of *D. stramonium* (including form *tatula*, form *stramonium*, and form *inermis*) exhibit similar seed lengths, ranging from 3.5 to 5 mm. Other species, such as *D. innoxia*, *D. metel*, and *D. ferox*, have slightly larger seeds, ranging from 5 to 7 mm. All the studied forms share a reniform (kidney-shaped) seed structure, a characteristic commonly associated with Datura species. Additionally, the mature seeds across all varieties are uniformly described as dark brown to black, ensuring consistency in seed color data across the different forms (**Figure 1**; **Supplementary Table 2**).

Pollen grain characteristics also exhibit variation among the species. *D. innoxia*, *D. metel*, and *D. ferox* have larger pollen grains, with polar axes ranging from 28 to 40 µm. In contrast, *D. stramonium form stramonium* and *D. stramonium form inermis* have pollen grains measuring between 23.3 and 34.8 µm, while *D. stramonium form tatula* has grains ranging from 26 to 32.2 µm. Despite these size differences, all species, including *D. ferox*, share a prolate-spheroidal pollen shape, which is typical of the genus. The larger pollen grains of *D. ferox* may contribute to enhanced reproductive success by improving fertilization chances under certain environmental conditions (**Supplementary Table 2**).

### 3.2 Molecular taxonomy

#### 3.2.1 Molecular characterization based on ISSR markers

**Figure 2:**
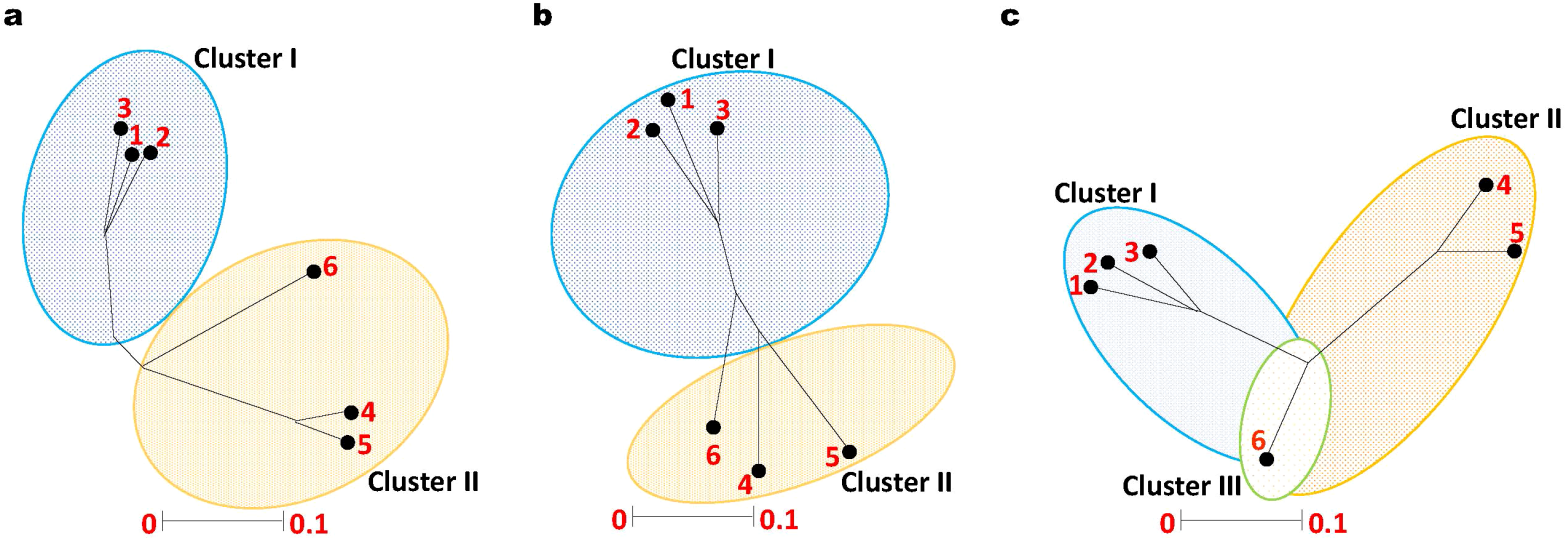
Unrooted Phylogenetic tree of Egyptian *Datura* genotypes based on (**a**). ISSR, (**b**). SCoT and (**c**). CDDP markers. The genetic similarity values varied between the six *Datura* genotypes which were labeled for clarity as follow; (1) *D. stramonium* form *stramonium*, (2) *D. stramonium* form *tatula*, (3) *D. stramonium* subsp. *inermis*, (4) *D. metel*, (5) *D. ferox* and (6) *D. innoxia*. The phylogenetic tree was constructed using Neighbor Joining method with Jukes-Camtor Models of Geneious 9.0.

Seeds of six distinct genotypes of *Datura* species, namely, *D. stramonium* form (*stramonium*, *tatula*, and *inermis*), *D. innoxia*, *D. metel*, and *D. ferox* were obtained from the National Genebank, Giza, Egypt illustrated in (**Supplementary Tables 1**, **2**) and (**Figures 1A–F**). It is worth mentioning that, these *Datura* accessions were collected from various environmental areas. Hence, it was important to explore the genetic variation between them. Therefore, we used ISSR markers to evaluate the genetic uniformity among the accessions. In this approach we used six ISSR primers (ISSR1, ISSR2, ISSR3, ISSR4, ISSR6 and ISSR8) to generate genetic markers (**Supplementary Figure 3**; **Supplementary Table 7**). Irrespective of the ratio, ISSR bands were identified as either nonexistent or existent. Every DNA locus was considered distinct. The analysis of variance test (**Supplementary Table 7**), indicated that there were highly significant differences between the six *Datura* genotypes. The six ISSR primers; exhibited an average of 108 fragments, 22 of which were monomorphic (**Supplementary Figure 3**; **Supplementary Table 7**). In contrast, 80 bands demonstrated polymorphism, with 62.92% polymorphism including 10 unique bands. For every primer, the average number of polymorphic ISSR markers was 8 fragments. The number of polymorphic bands varied between 9 and 18, while the molecular size varied between 150 and 1500 bp. Out of the polymorphic bands generated using the ISSR primers, we were successful in identifying and contrasting the molecular genetic variations between the studied *Datura* genotypes (**Supplementary Table 8**). Furthermore, these molecular genetic variations are regarded as fundamental taxonomic distinctions amongst the investigated *D*atura accessions. The ISSR primers that produced species specific marker; three in *D*. *stramonium* form *inermis*, three positive markers in genotypes *D*. *stramonium* form *stramonium*, form *tatula*, four in *D*. *metel*, and three in *D. ferox*. Pairwise comparisons between the six *Datura* genotypes varied from (0.87 to 0.37), with an average of (0.53), with the highest level of genetic similarity among the genotypes (*D. stramonium* form *stramonium* and form *tatula*), followed by the genotypes form *stramonium* and form *inermis* (0.85). The genotypes with the lowest rank of similarity *D. stramonium* form *inermis* and *D. ferox* (0.37). The genotypes of form *tatula* and form *inermis* shared a genetic similarity value of 0.75. In addition, comparison between the genotypes of form *stramonium* and *D. metel*, form *inermis* and *D. metel*, form *stramonium* and *D. innoxia* and form *tatula* and *D. metel* showed a range of genetic similarity ratio of 0.59, 0.52, 0.514, and 0.51 respectively. The remaining genetic similarity values proved to be low to medium in this respect. The results of the phylogenetic analysis via unrooted tree (**Figure 2a**) separated all *Datura* genotypes into two main branches: branch I contained the genotypes *D. innoxia* and *D. ferox*, while branch II is divided into two sub-branches: the first contained *D. stramonium* form. *metel*, and the second sub-branch is divided into two groups the first group included the genotypes *D. stramonium* form. *stramonium* and form. *Tatula*, while the second group included only *D. stramonium* form *inermis*.

#### 3.2.2 Molecular characterization based on SCoT markers

To further examine the genetic uniformity, we employed another evaluation methodology using SCoT markers. To this end, six primers (SCoT1, SCoT2, SCoT11, SCoT12, SCoT20 and SCoT22) were employed to generate the banding patterns (**Supplementary Table 10**). The analysis of variance test results (**Supplementary Table 10**), indicated that there were highly significant differences between the six *Datura* genotype features that were investigated. (**Supplementary Table 10**). The six SCoT primers exhibited an average of 53 fragments, 17 of which were monomorphic. In contrast, 36 bands demonstrated polymorphism, with 58.6% polymorphism including 5 unique bands. For every primer, the average number of polymorphic SCoT markers was 8.8 fragments. The number of polymorphic bands varied between 5 and 7, while the molecular size varied between 230 and 1780 bp **(Supplementary Table 10**). Two separate branches were formed by grouping the six *Datura* genotypes using the unweighted neighbor-joining approach (**Figure 2b**). Branch I comprised the three genotypes *D. stramonium* form *stramonium*, *D*. *stramonium* form *tatula* and *D. stramonium* form *inermis*. Branch II contained the remaining genotypes *D. innoxia*, *D. ferox* and *D. metel*.

#### 3.2.3 Molecular characterization based on CDDP markers

**Figure 3:**
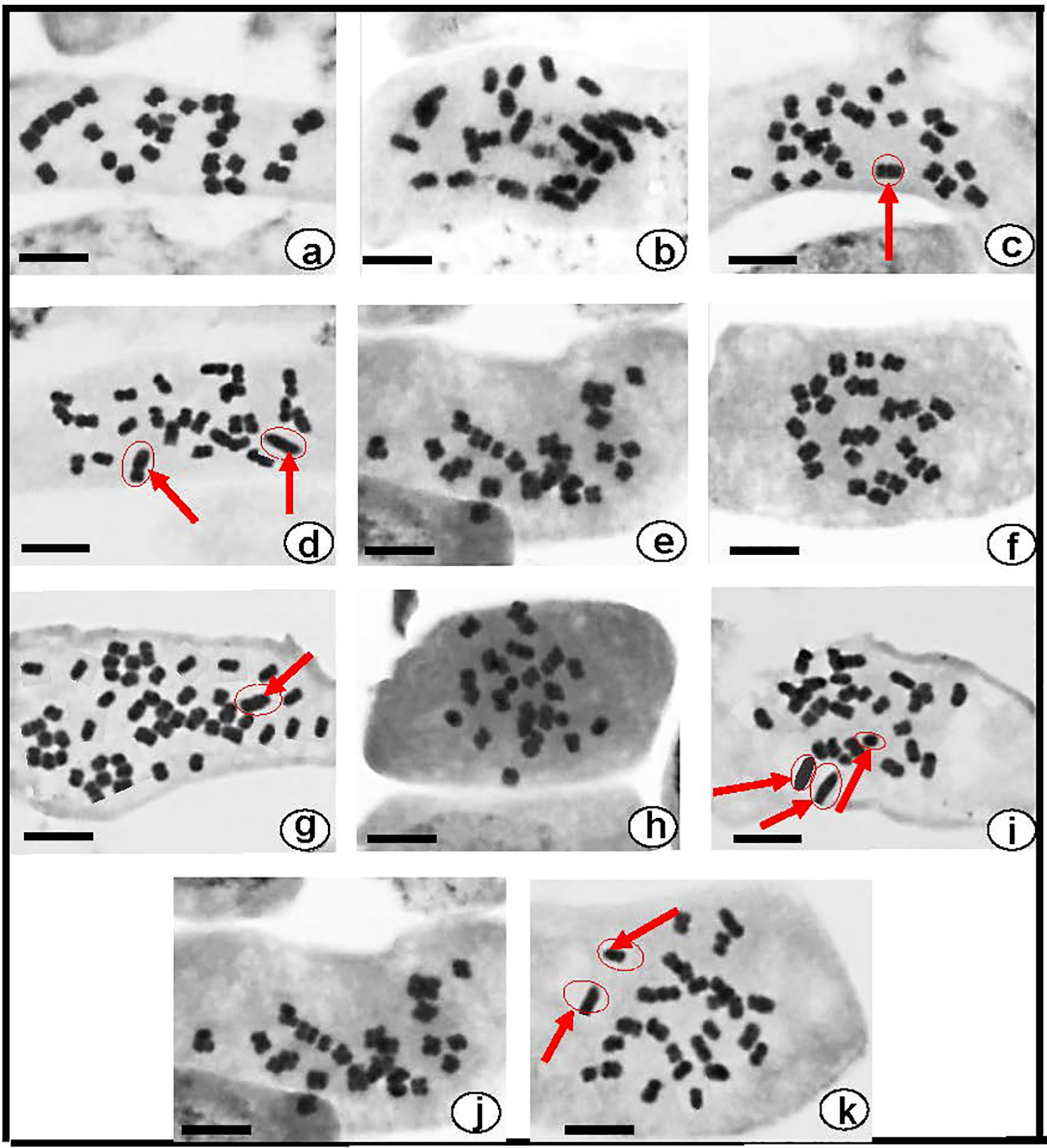
Somatic metaphase chromosomes of six Egyptian *Datura* genotypes **(a)**. *D. stramonium* form *stramonium* (2n = 24), (**b–d)**. *D. stramonium* form *tatula* (2n = 24, 25 and 26), **(e)**. *D. stramonium* subsp. *inermis* (2n = 24), **(f, g)**. *D. metel* (2n = 24 and 48) **(h, i)** . *D. ferox* (2n = 24 and 27), **(j, k)** . *D. innoxia* (2n = 24 and 27). Red arrows refer to the alterations in size of certain somatic chromosomes. Bar: 10*f*Êm.

The six *Datura* genotypes were subjected to fingerprinting using CDDP primers. A total of 105 bands, of which 77 were polymorphic, were produced (**Supplementary Table 11**). With an average of 6.4 polymorphic bands per primer, the range of numbers varied from 4 ERF1R and MYB1F to 9 PR1-1F and MYB1R. The percentage of polymorphism varied from 33.3% to 75%, with an average polymorphism of 52.3% for all genotypes. PIC values varied from 0.13 (for MYB1F and ERF1R) to 0.46 (for PR1F and MYB1R), with an average of 0.30 for each primer (**Supplementary Table 11**). Three separate branches were formed by grouping the six *Datura* genotypes using the unweighted neighbor-joining approach (**Figure 3c**). Branch I comprised the three genotypes *D. stramonium* form. *stramonium*, *D*. *stramonium* form. *tatula* and *D. stramonium* form *inermis*. Branch II contained the genotypes *D. innoxia* and *D. ferox*. The remaining genotype *D. metel* formed a third branch, which intersects with branch I and II (**Figure 2c**).

#### 3.2.4 The efficiency of ISSR, SCoT and CDDP marker systems to reveal the genetic diversity among *Datura* accessions

Given the high polymorphism percentage and PIC values in *Datura* genotypes, the current study demonstrated that all marker systems proved useful in evaluating genetic diversity. Generally, PIC and polymorphism values for CDDP markers were greater than those for ISSR and SCoT markers. A satisfactory fit for clustering was suggested by the cophenetic coefficient for all marker systems (ISSR = 0.67, SCoT = 0.76, and CDDP = 0.81). A positive correlation (*r* = 0.64*) was observed between the marker systems according to the mantel test correlation values (**Figure 2**).

#### 3.2.5 Chromosome numbers and karyomorphology

All of the *Datura* genotypes tested had the same somatic chromosomal number, 2n=2x=24 although some aneuploidies and polyploid occurred in certain *Datura*. In *D. stramonium* form *stramonium* (**Figure 3a**) and form *inermis* (**Figure 3e**) had a chromosomal number of 2n=24 in all of the plants examined, and no evidence of a satellite chromosome (SAT-Chr.). The chromosomal number of *D. stramonium* form *tatula* varied between 2n=24, 25, and 26 (**Figures 3b–d**). The observed aneuploidy was caused by the insertion of one or two additional copies of chromosomes 8 and 9. (2n=2x+1 = 25 or 2n=2x+2 = 26). For *D. metel*, the chromosomal number varied from 24 and 48 chromosomes (**Figures 3f, g**), with polyploidy having four complete sets of chromosomes (4n=4x=48) (**Figure 3g**). The chromosomal study of *D. innoxia* and *D. ferox* revealed that both contain 24 chromosomes in the normal form (**Figures 3h, j**) and 27 chromosomes in the abnormal forms. These chromosomal aneuploidies come from the insertion of three extra copies of chromosomes 7, 8, and 9 (2n=2x+3 = 27) (**Figures 3i, k**). Regarding karyomorphology, no alterations were seen in the size of the somatic chromosomes or the satellite chromosome for all six genotypes investigated in their normal forms (**Figures 3a, b, e, f, h, j**). Conversely, alterations in the size of certain somatic chromosomes and rarely small dot-shaped satellite were seen in the abnormal forms of *D. stramonium* form *tatula* (**Figures 3i, k**), *D. metel*, *D. innoxia*, and *D. ferox* (**Figures 3c, d, g, i, k**).

### 3.3 Chemical taxonomy

#### 3.3.1 Diversity between *Datura* genotypes of total phenolic content

**Figure 4:**
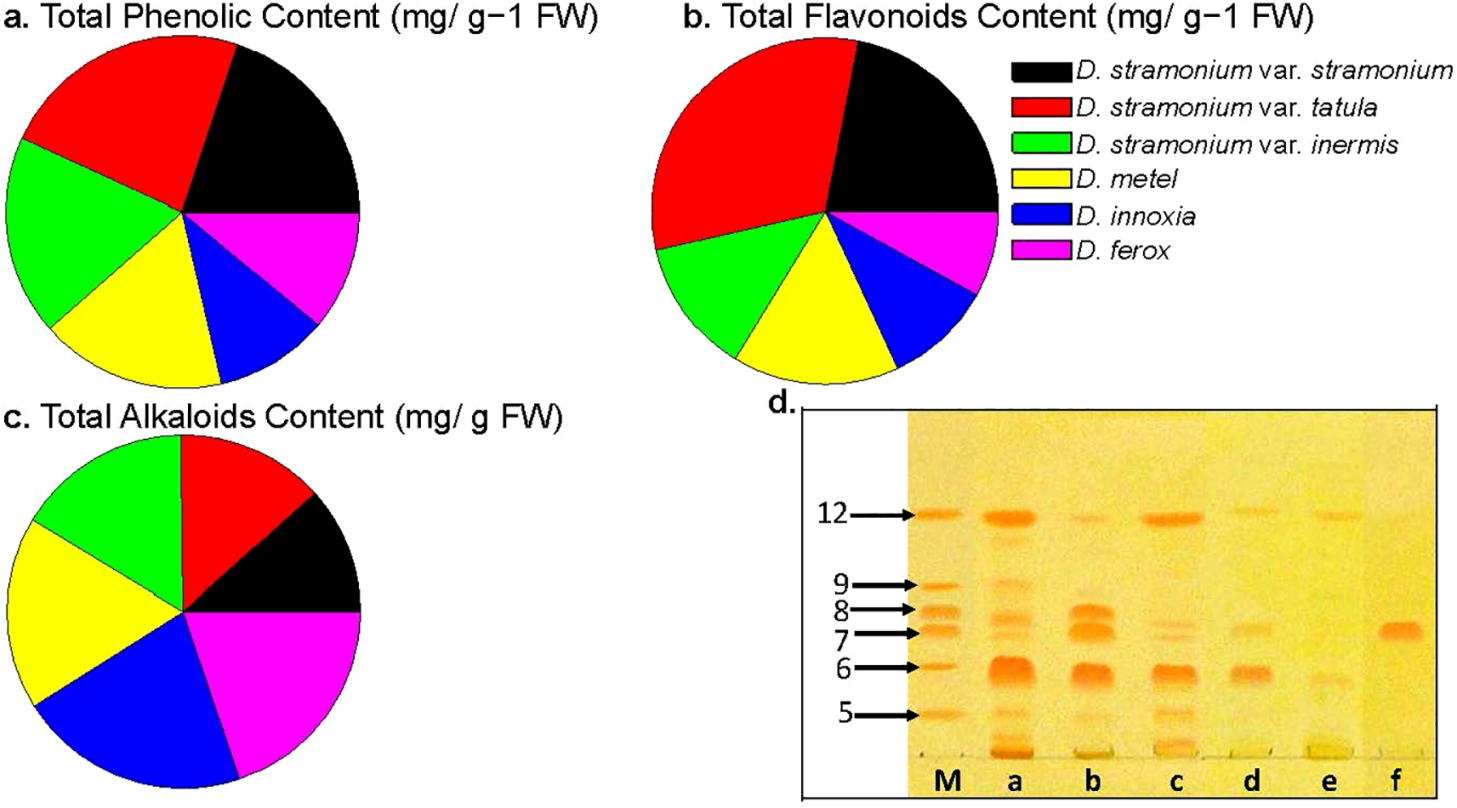
Genetic diversity between *Datura* genotypes affects secondary metabolites accumulation. **(a)**. Total phenolic content expressed as mg of gallic acid equivalent (GAE) per g of dried extract, (**b)**. Total flavonoid content using quercetin equivalent. and (**c)**. Total alkaloid content was expressed as mg of AE per g of extract. The data shown are the mean ± SE (n = 3). **(d)**. Chromatogram of alkaloid fractions from: (a) *D. stramonium* form *stramonium* roots, (b) *D. stramonium* form *tatula* roots, (c) *D. stramonium* subsp. *inermis* roots, (d) *D. metel* roots, (e) *D. ferox* roots and (f) *D. innoxia* roots. (m) standards include hyoscyamine, scopolamine, hyoscyamine, scopoletin, atropine and cuscohygrine. (HPTLC Si60 F254 (Merck, Germany) – chloroform:methanol:acetone:25% ammonia (75:15:10:1.8 - v/v/v/v); plates were developed twice – 4.0 cm first run, 3.0 cm second run.) The photography was acquired after derivatization with Dragendroff’s reagent.

Comparative phytochemical analyses of fresh *Datura* leaves showed a total phenolic content ranging from 0.87 to 1.96 mg/g^−1^ FW with values of 1.96, 1.67 and 1.54 mg/g^−1^ FW, respectively, *D. stramonium* form *tatula*, *stramonium* and *inermis* had the highest phenolic contents. Opposed to this, *D. innoxia*, *D. ferox*, contained the lowest phenolic levels, with values of 0.87, 0.93, and 1.12 mg/g^−1^ FW, respectively. Notably, the total phenolic content of *D. metel* (1.44 mg/g^−1^ FW) in the current study exhibited intermediate levels across all genotypes (**Figure 4a**).

#### 3.3.2 Diversity between *Datura* genotypes of flavonoid content

As anticipated, the total flavonoid accumulation rate in *Datura* fruits was correlated with the total phenolic content. Compared to other genotypes, the total flavonoid content of *D. stramonium* form *tatula* fruits had the greatest flavonoid value (up to 0.75 mg/g^-1^ FW) among all studied *Datura* genotypes, followed by *D. stramonium* form *stramonium* and inermis (up to 0.52 and 0.30 mg/g^-1^ FW), respectively. Conversely, both of *D. ferox* and *D. innoxia* had the lowest values (up to 0.19 and 0.24 mg/g^-1^ FW), respectively. Notably, the total flavonoid content of *D. metel* (0.30 mg/g^−1^ FW) in the present study exhibited intermediate levels across all genotypes (**Figure 4b**).

#### 3.3.3 Diversity between *Datura* genotypes of total alkaloid content

Analysis of total alkaloid contents showed that *D. innoxia* had the highest total alkaloid content (up to 103.21 ± 5.9 mg/g), followed by *D. ferox* and *D*. *stramonium* form *inermis* (up to 96.13 ± 4.3 and 95.67 ± 6.4 mg/g), respectively (**Figure 4c**), while *D. stramonium* form *stramonium*, *tatula* and *inermis* had the lowest total alkaloid concentrations (up to 56.66 ± 3.3, 65.85 ± 4.5 and 78.22 ± 4.2 mg/g), respectively. It is interesting to observe that *D. metel*’s total alkaloid content reached an intermediate value reaching (85.67 ± 5.2 mg/g) when compared to the other investigated genotypes.

#### 3.3.4 Qualitative analysis via HPTLC for tropane alkaloids in the extracts of *Datura* genotypes

Using HPTLC Si60 F254 plates prerequisite with mobile phase vapors (chloroform: methanol: acetone: 25% ammonia percentages of 75:15:10:1.8, v/v/v/v), complete separation across all compounds was achieved. In a Camag twin trough chamber, the chromatograms were produced twice (at intervals of 4.0 and 3.0 cm) and visualized using Dragendorff’s reagent. *Datura* samples were subjected to quantitative analysis using densitometric detection (λ = 190 and 520 nm). For hyoscyamine, scopolamine, hyoscyamine, scopoletin, atropine and cuscohygrine, the calibration curves were linear and fell between 500-4,000 ng and 500 and 2,000 ng, respectively. Overall, six tropine alkaloid compounds including scopolamine, scopoletin, atropine, hyoscyamine, cuscohygrine and anisodamine (bands no. 12, 9, 8, 7, 6 and 5 respectively), were observed within the extracts of *Datura* genotypes (**Figure 4d**). The phytochemical examination of alkaloids in *D. stramonium* forms (*stramonium*, *tatula*, and *inermis*) contains all alkaloids except scopoletin, that is not found in *D. stramonium* form *inermis*. Interestingly, the genotype *D. innoxia* contained a single band of the alkaloid hyoscyamine only, followed by *D. ferox* which had two compounds including scopolamine and cuscohygrine. In the case of *D. metel*, the alkaloids scopolamine, atropine and anisodamine were absent, while scopolamine, hyoscyamine and cuscohygrine were present (**Figure 4d**). Chromatogram analysis of alkaloid compounds clearly indicated chemical diversity among *Datura* genotypes in the present study.

#### 3.3.5 GC-MS investigation leads to novel tropane alkaloids discovery in *Datura* genotypes

**Figure 5:**
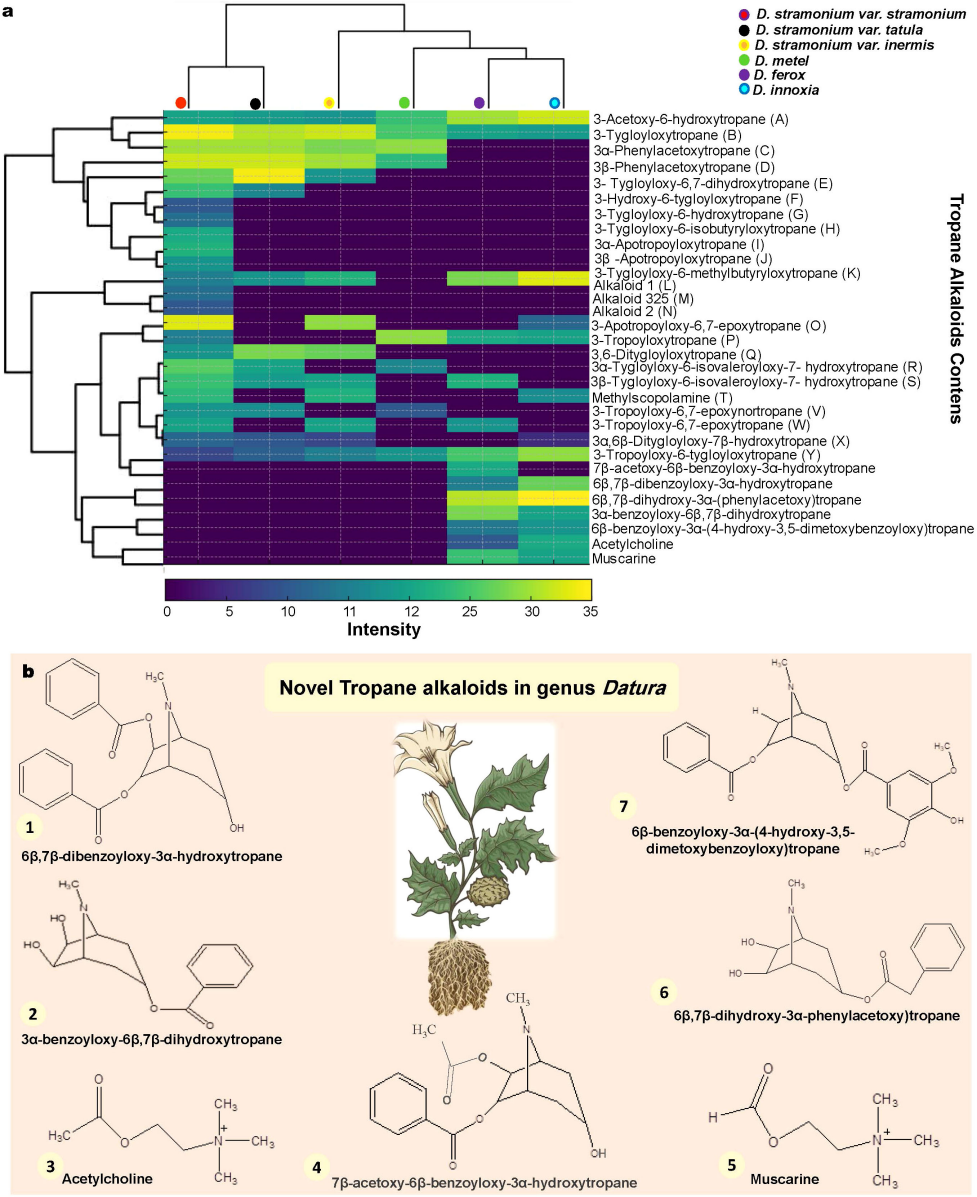
GC-MS investigation leads to novel TAs discovery in *Datura* genotypes for the first time in this genus. **(a)**. Heatmap analysis of 31 identified TA compounds between the six Egyptian *Datura* genotypes. The lowest ratios are in blue, and the highest ratio in yellow. (**b)**. Seven novel TAs were discovered for the first time in the genus *Datura* including 1. 6β,7β-dibenzoyloxy-3α-hydroxytropane, 2. 3α-benzoyloxy-6β,7β-dihydroxytropane, 3. Acetylcholine, 4. 7β-acetoxy-6β-benzoyloxy-3α-hydroxytropane, 5. Muscarine, 6. 6β,7β-fdihydroxy-3α-phenylacetoxy)tropane, 7. 6β-benzoyloxy-3α-(4-hydroxy-3,5-dimetoxybenzoyloxy)tropane. .

The results of Gas chromatography mass spectrometry reported thirty-one TAs. According to Philipov S and Berkov S. (2002) (Philipov and Berkov, 2002), they are all characterized by the tropane nucleus ions at m/z 81, 82, 83, 94, 95, 96, 113 and 124. The identities of alkaloid 1 (L) and alkaloid 2 (N) were unknown (**Figure 5a**; **Supplementary Table 12**). In GC-MS, alkaloids C, D, I, J, R, S and X show up as twin peaks with the same mass spectra. According to Witte et al. (1987) (Witte et al., 1987), they are isomeric tropine and pseudotropine esters. Since the stereochemistry of 3α-Phenylacetoxytropane (C), 3β-Phenylacetoxytropane (D), 3α-Apotropoyloxytropane (I), 3β-Apotropoyloxytropane (J), 3α-Tygloyloxy-6-isovaleroyloxy-7- hydroxytropane (R), 3β-Tygloyloxy-6-isovaleroyloxy-7-hydroxytropane (S) and 3α,6β-Ditygloyloxy-7β-hydroxytropane (X) could not be determined only from MS data, it was not covered. The TAs 3-Acetoxy-6-hydroxytropane (A), 3-Tygloyloxytropane (B) and 3-Tropoyloxy-6-tygloyloxytropane (Y) were common compounds among all *Datura* genotypes in the present study. Interestingly, the tropine alkaloids A, B, C, D, E, H, L, O and Q were common compounds between the three *D. stramonium* forms *stramonium*, *tatula* and *inermis*, indicating a chemical similarity that is compatible with genetic relationships. Similarly, the presence of alkaloids A, B, I, L, P and Y as well as the lack of alkaloids C, D, F, H, K, M, N, Q, R and V in the genotypes *D. innoxia* and *D. ferox* suggests chemical similarity. Interestingly, the TAs 3-Tygloyloxy-6-methylbutyryloxytropane (K), alkaloid 325 (M) and alkaloid 2 (N) are unique to *D. stramonium* form *stramonium*. In the same manner, the TAs 3-Tropoyloxy-6-tygloyloxytropane (Y), 7β-acetoxy-6β-benzoyloxy-3α-hydroxytropane, 6β,7β-dibenzoyloxy-3α-hydroxytropane, 6β,7β-dihydroxy-3α-(phenylacetoxy) tropane, 3α-benzoyloxy-6β,7β-dihydroxytropane, 6β-benzoyloxy-3α-(4-hydroxy-3,5-dimetoxybenzoyloxy) tropane, Acetylcholine and Muscarine are unique bands for both of *D. innoxia* and *D. ferox*. Furthermore, *D. metel* seemed to be chemically in the middle of all studied *Datura* genotypes (**Figure 5a**; **Supplementary Table 12**). Interestingly, novel TAs were identified in both of *D. innoxia* and *D. ferox* as 7β-acetoxy-6β-benzoyloxy-3α-hydroxytropane, 6β,7β-dibenzoyloxy-3α-hydroxytropane, 6β,7β-dihydroxy-3α-(phenylacetoxy) tropane, 3α-benzoyloxy-6β,7β-dihydroxytropane, 6β-benzoyloxy-3α-(4-hydroxy-3,5-dimetoxybenzoyloxy) tropane, Acetylcholine and Muscarine (**Figure 5b**; **Supplementary Table 12**). Fortunately, these metabolites have a number of pharmacological activities that include antibacterial, antimicrobial, antiproliferative, proaptotic, hepatoprotective, antihypertensive and anticancer (da Silva et al., 2021). Notably, *D. stramonium* form *stramonium* contained more TAs (24 compounds) than other genotypes. The overall number and concentration of compounds were similar in *D. inoxia* and *D. ferox*. Interestingly, TA compounds observed in *D. stramonium* form *inermis* and form *tatula* were distributed between D. *stramonium* form *stramonium*. Conversely, *D. metel* has the lowest number and concentration of TAs.

The GC-MS TAs data yielded a dendrogram that divided the six *Datura* genotypes into two separate clusters (**Figure 5a**). The Dice-similarity scores ranged from 0 to 5. The first cluster (I)

contained both of *D. stramonium* form *stramonium* and *D. stramonium* form *tatula*, whereas the second cluster (II) was separated into two sub-clusters, The first sub-cluster had just *D. stramonium* form *inermis*, a monotypic variant, whereas the second sub-cluster was split into two groups, the first group had only *D. metel*, whereas the second group included the remaining two genotypes, *D. innoxia* and *D. ferox* (**Figure 5a**).

#### 3.3.6 UV spectroscopy in conjunction to multivariate data analysis

**Figure 6:**
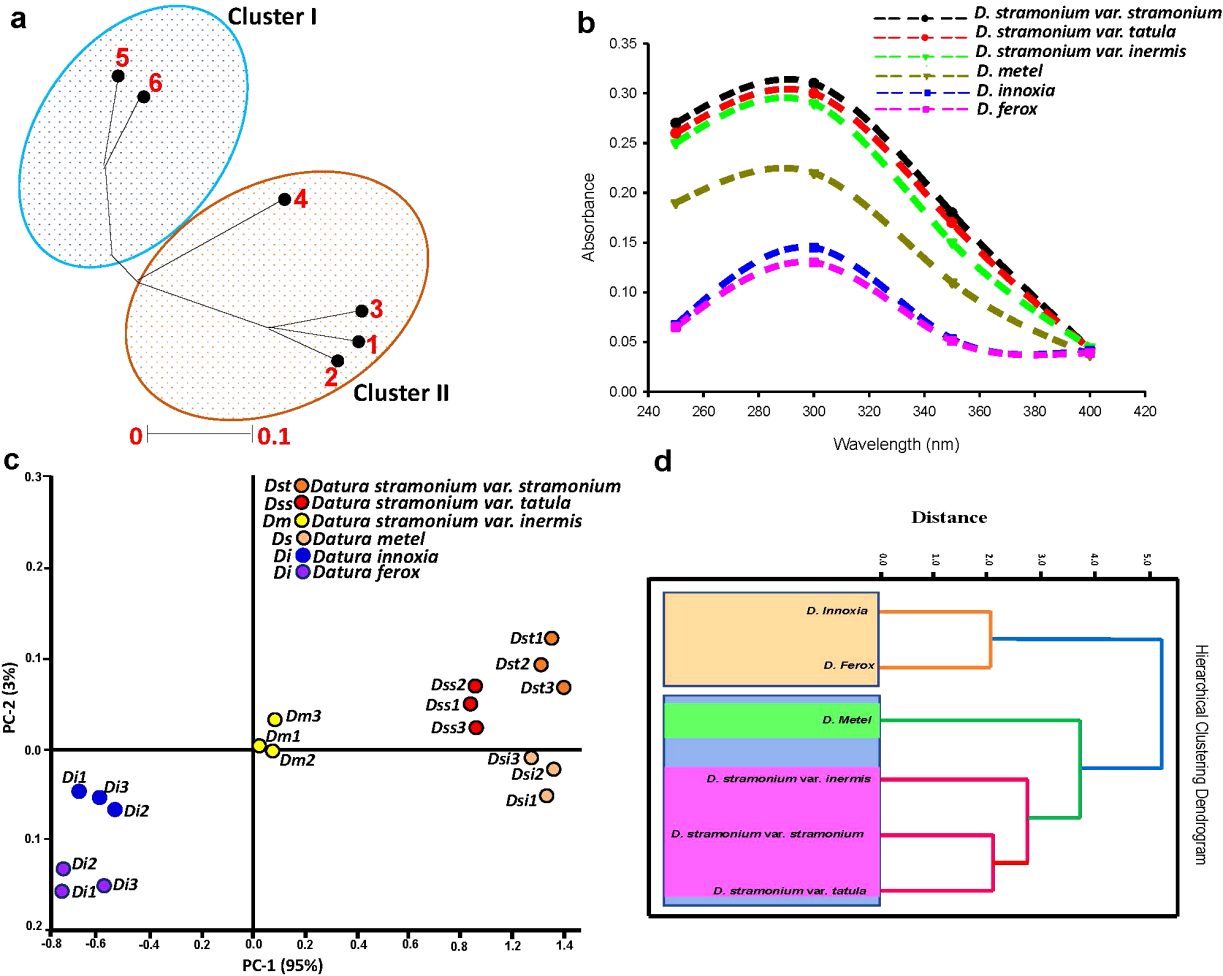
Evaluation of genetic variability between Egyptian *Datura* genotypes. **(a)**. Unrooted phylogenetic tree based on the GC-MS TAs data. The genetic similarity values varied between the six *Datura* genotypes which were labeled for clarity as follow; (1) **(*D*)** *stramonium* form *stramonium*, (2) (*D*) *stramonium* form *tatula*, (3) *D. stramonium* subsp. *inermis*, (4) *D. metel*, (5) *D. ferox* and (6) *D. innoxia*. The phylogenetic tree was constructed using Neighbor Joining method with Jukes-Camtor Models of Geneious 9.0. (**b)**. UV spectroscopic profiling coupled to unsupervised chemometric techniques. UV spectra of the studied *Datura* genotypes. (**c)**. UV spectroscopic profiling coupled to unsupervised chemometric techniques. Principal Components Analysis (PCA) score plot of the studied *Datura* genotypes. **(d)**. UV spectroscopic profiling coupled to unsupervised chemometric techniques. Hierarchical Cluster Analysis (HCA) score plot of the studied *Datura* genotypes. .

Unrooted phylogenetic tree that was generated from integrated total phenolics, flavonoids and alkaloids information divided the six *Datura* genotypes into two main branches (**Figure 6a**), with Dice-similarity scores fluctuating between 0 to 0.1. The first branch (I) had both of *D. ferox* (5) and *D. innoxia* (6). Branch (II) divided into two sub-branches, the first sub-branch only had *D. metel*, whereas the second sub-branch contained two groups, the first group included *D. stramonium* form *stramonium* and *D.* stramonium form *tatula*, while the other group contained *D. stramonium* form *inermis*.

UV spectroscopic assessment of six *Datura* genotypes was carried out to confirm genetic and chemical taxonomy patterns (**Figure 6b**). Principal component analysis (PCA) plot was used to find changes in UV absorption patterns across *Datura* genotypes. PC1 and PC2 accounted for 98% of the variation within genotypes. The PCA score plot (**Figure 6c**) revealed genotypes that are substantially similar. According to PC1 (which accounts for 95% of variability among genotypes), *D. stramonium* form *stramonium*, *D. stramonium* form *tatula*, and *D. stramonium* form *inermis* were on the positive side, whereas *D. ferox* and *D. innoxia* were on the negative side. Interestingly, *D. metel* was on the positive side, close to the negative side. *D. stramonium* form *stramonium*, form *tatula*, and form *inermis*, as well as *D. metel*, were classed as “outliers” and seen on the positive side, whilst *D. ferox* and *D. innoxia* were observed on the negative side. This conclusion may be drawn based on the total phenolic, flavonoids, alkaloids, and individual contents, since the genotypes of each group had nearly same levels (**Figure 6a**), and showed significant clustering within phylogenetically related genotypes. The six genotypes were separated into two main clusters depending on the HCA dendrogram: the first cluster (I) was divided into two sub-clusters. The first sub-cluster was split into two groups: the first group included *D. stramonium* form *stramonium* and form *tatula*, and the second group only included D. *stramonium* form *inermis*. The second sub-cluster had just *D. metel*, a monotypic variation, whereas the second cluster (II) featured the remaining two genotypes, *D. innoxia* and *D. ferox* (**Figure 6d**).

### 3.4 The impact of transcript variation in tropane alkaloid biosynthesis genes on *Datura* biodiversity

**Figure 7:**
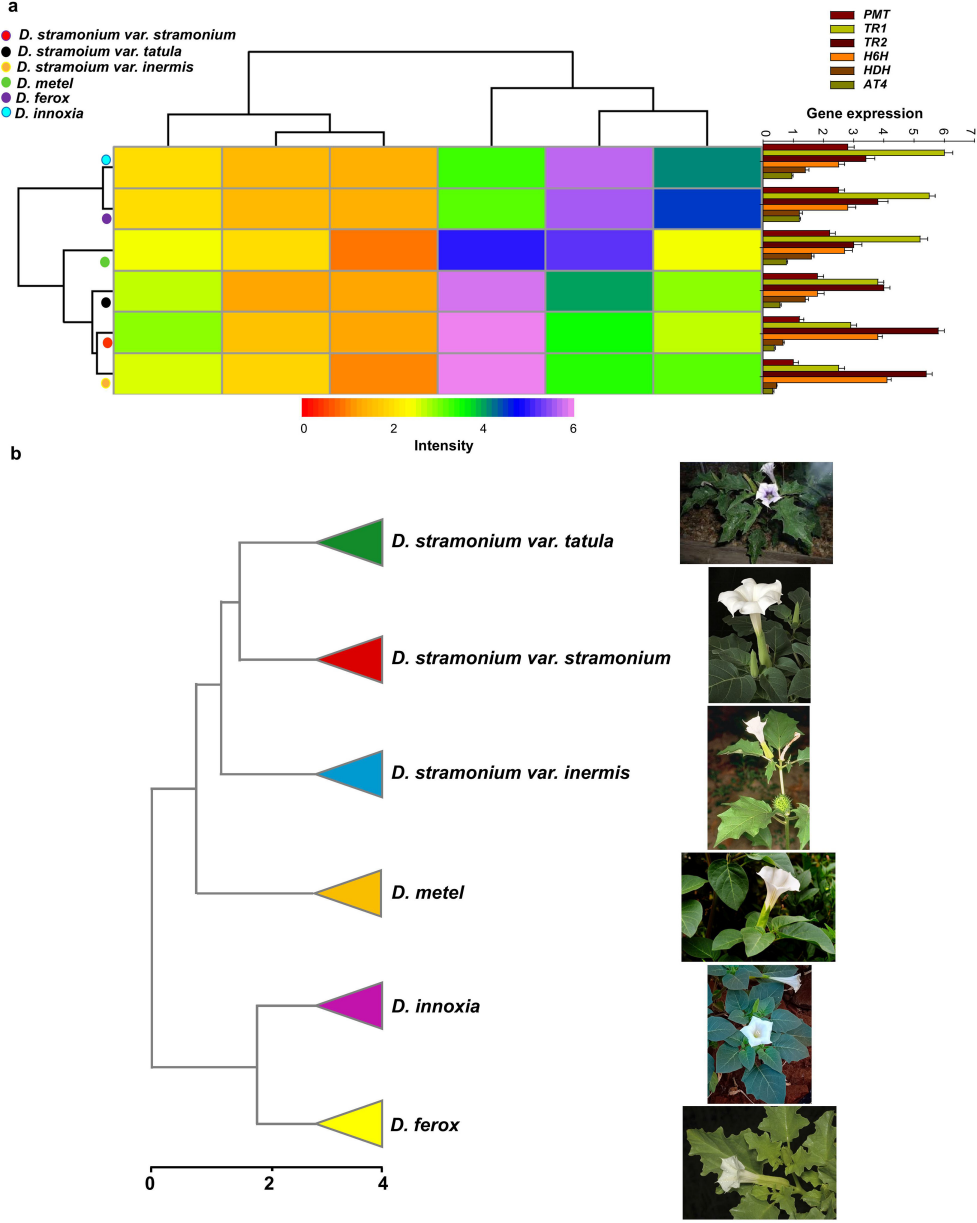
Transcripts of biosynthesis genes affects TAs accumulation hence influence on *Datura* biodiversity. **(a)**. Quantitative expression analysis of TA biosynthetic genes in leaves of *Datura* genotypes. *putrescine N-methyltransferase* (*PMT*), *tropinone reductase I* (*TR1*), *tropinone reductase II* (*TR2*), *aromatic amino acid aminotransferase 4* (*AT4*), *hyoscyamine dehydrogenase* (*HDH*), and *hyoscyamine 6 beta-hydroxylase* (*H6H*). The data shown are the mean ± SE (n = 3). (**b)**. UPGMA dendrogram depicting rearrangement of the six Egyptian *Datura* genotypes based on morphological, molecular and chemical investigations.

Six biosynthetic genes with identical primer sequences were found in the genomes of the six *Datura* genotypes, out of ten essential genes involved in the TA synthesis pathway. The transcription level of genes that encode *putrescine N-methyltransferase* (*PMT*), *tropinone reductase I* (*TR1*), *tropinone reductase II* (*TR2*), *aromatic amino acid aminotransferase 4* (*AT4*), *hyoscyamine dehydrogenase* (*HDH*), *hyoscyamine 6 beta-hydroxylase* (*H6H*) were confirmed using quantitative real-time PCR (qRT-PCR). Interestingly, all six genes were expressed differently in the leaves of the six *Datura* genotypes, with the *TR1* gene being the most expressed in the leaves of all studied genotypes, followed in order by *TR2, H6H, PMT, HDH*, and *AT4* (**Figure 7a**). In detail, the expression levels of *PMT*, *TR1* and *AT4* genes were elevated gradually up to (1.0, 1.2, 1.8, 2.2, 2.5 and 2.8), (2.5, 2.9, 3.8, 5.2, 5.5 and 6.0) and (0.32, 0.37, 0.56, 0.77, 1.2 and 0.9), respectively in the leaves of *D. stramonium* form *inermis*, *D. stramonium* form *stramonium, D. stramonium* form *tatula, D. metel, D. ferox* and *D. innoxia*, respectively (**Figure 7a**). In contrast to the order of *PMT*, *TR1* and *AT4*, the transcripts of *H6H* and *TR2* genes were induced rapidly up to (2.5, 2.8, 2.7, 1.8, 3,8 and 4.1) and (3.4, 3.8, 3.0, 4.0, 5,8 and 5.4) in leaves of *D. innoxia*, *D. ferox, D. metel, D. stramonium* form *tatula, D. stramonium* form *stramonium* and *D. stramonium* form *inermis*, respectively. Remarkably, the transcriptional levels of *HDH* appeared to vary oscillatingly and randomly up to (1.4, 1.2, 1.6, 1.4, 0.66 and 0.44), respectively in *D. innoxia, D. ferox, D. metel, D. stramonium* form *tatula, D. stramonium* form *stramonium* and *D. stramonium* form *inermis*, respectively (**Figure 7a**). For further confirmation, the expression profiles of the remaining genes involved in TA synthesis including *Ornithine decarboxylase* (*ODC*), *N-methylputrescine oxidase* (*MPO*)*, type III polyketide synthase* (*PYKS*), *tropinone synthase* (*CYP82M3*)*, phenylpyruvic acid reductase* (*PPAR*), *phenyllactate UDP-glycosyltransferase* (*UGT1*) and *littorine mutase* (*CYP80F1*) were investigated. All these genes were expressed and estimated except *PPAR* and *CYP80F1* genes which were not detected in all investigated Datura genotypes. Interestingly, the expression levels of *ODC* and *MPO* were significantly higher in both *D. ferox* and *D. innoxia* compare to the remaining genotypes. Remarkably, the transcriptional levels of *PYKS, CYP82M3* and *UGT1* appeared to vary oscillatingly and randomly in all examined genotypes (**Supplementary Figure 4**). These findings were supported by the dendrogram that was yielded based on expression level of TA biosynthetic genes along with the above-mentioned results regarding phytochemical and molecular analysis. The dendrogram divided the six *Datura* genotypes into two separate clusters (**Figure 7b**). The Dice-similarity scores ranged from 0 to 4. The first cluster (I) divided into two sub-clusters, the first sub-cluster separated to two groups, the first group contained both of *D. stramonium* form *stramonium* and form *tatula*, whereas the second group contained had just *D. stramonium* form *inermis*, a monotypic variant. The second sub-cluster contained only *D. metel*, whereas the second main cluster (II) included the remaining two genotypes, *D. innoxia* and *D. ferox* (**Figure 7b**).

## 4 Discussion

Drug development methodologies and enhanced therapeutic usage in the genus *Datura* rely substantially on a thorough understanding of the species’ molecular and phytochemical variability (John et al., 2014). Several *Datura* species grows in both Egypt and the Kingdom of Saudi Arabia, both in urban and rural locations. It spreads profusely over these two nations. These two countries’ flora overlapped due to their shared borders and geographical proximity (Al-Shaikh and Sablay, 2005). The current study aims to clarify the importance of identifying phylogenetic relationships among specific *Datura* genotypes within Egyptian flora via contemporary molecular taxonomy techniques, counts of chromosome numbers and karyomorphology, as well as chemical taxonomy along with the UV spectroscopic pattern. To date, this is the first study to identify the evolutionary relationship of the genus *Datura* by combining the capabilities of molecular and chemical taxonomy. Additionally, novel TAs in the genus *Datura* were described for the first time. According to the results of recent study, the quantity of TAs and the expression level of its biosynthetic genes were determined in the leaves of *Datura* genotypes under investigation. Integrating genetic and chemical taxonomy discoveries with previous findings on the morphological taxonomy of genus *Datura* can help establish an appropriate biosystematic classification of the evaluated genotypes (Ewas, 2023).

*Datura* genotypes are global plants with phenotypic plasticity in bloom color (white or violet) and capsule shape (spiny or smooth). According to Blumer (1996) (Blumer, 1996), variations within the genus are widespread over a geographic spectrum and can be attributed to genetic or chemical variations across distinct populations at various locales. The taxonomic identification of *Datura* genotypes is currently being debated across the world. Furthermore, taxonomists did not establish the actual taxonomic position of these genotypes in Egypt (Hassan et al., 2019). Of the many recognized species and varieties of *Datura* genotypes only six genotypes are currently grown in Egypt including *D. stramonium* form *stramonium*, *D. stramonium* form *inermis*, *D. stramonium* form *tatula*, *D. metel*, *D. ferox*, and *D. innoxia* (Täckholm, 1974; Boulos, 2002; Boulos, 2009; Hassan et al., 2019; Rabei et al., 2019). Twenty-four macro- and micromorphological characteristics served as the basis for taxonomic evaluation. Along with respect to flower and fruit dimensions, the most significant physical characteristics that made the six genotypes clearly recognizable were flower and stem color. Except for *D. stramonium* form *tatula*, which has violet flowers, all of the genotypes examined have white flowers dense and uneven spines were seen in the capsules of all genotypes. These findings supported by those of (Hassan et al., 2019) and (Avery, 1959), who asserted that the six *Datura* genotypes studied have these characteristics. In contrast to Dave et al. (1980) (Dave,. et al., 1980), who asserted that *D. stramonium* possessed exclusively multicellular, non-glandular trichomes, our data showed the existence of glandular and non-glandular trichomes in both of *D. stramonium* form *stramonium* and *D. stramonium form tatula.* Conversely, the leaf examination of *D. stramonium* form *inermis* indicated the absence of all trichome types, while SEM for each of *D. metel, D. ferox* and *D. innoxia* showed only eglandular trichomes. In same manner, anisocytic stomata, are present in all genotypes. This variation in trichome type may influence the plants’ defense mechanisms against herbivores and environmental stressors (Ewas et al., 2016; Ewas et al., 2017).

According to cytogenetic analyses of the Egyptian *Datura* genotypes, all the six genotypes had a diploid chromosome number (2n=2x=24), with the exception of a few aneuploidies and polyploid. Certain *Datura* genotypes, including *D. Stramonium* form *tatula, D. innoxia*, and *D. ferox*, were polyploid (2n=2x+1 = 25, 2x+2 = 26, 2x+3 = 27, 2x+4 = 28), while, *D. metel* was polyploid (2n=4x=48). Similar findings were made previously by Bonicke (1911) (Bonicke, 1911; Badr, 2022), who said that *D. tatula* and *D. stramonium* share a haploid chromosome number of 12. The same findings were reported by several investigations, including Satina (1959), Fedorov (1969), Tutin et al. (1976) and Goldblatt (1981) (Satina, 1959; Fedorov, 1969; Tutin et al., 1976; Goldblatt, 1981). Some mild aneuploidies in chromosomes 8 and 9 (2n=2x+2 = 26) were reported by Hassan and Amer (2019) (Hassan et al., 2019). According to Prota (2016) (PROTA, 2016), chromosomes. Variants of *D. stramonium* having chromosomal numbers of 2n = 12, 25, 26, 36, or 48 have been reported. The karyotyping results showed that the six genotypes under study had some different chromosomal morphologies in the abnormal forms. Considering the relative chromosomal lengths of the genotypes under study, several significant variations were discovered in the abnormal forms. The observed differences indicated the genetic diversity within the genus *Datura*. Our results agreed with the findings of Prota (2016) (Hassan et al., 2019) and Hassan and Amer (2019) (PROTA, 2016).

Integrating the findings of modern molecular taxonomy methods with chemical taxonomy, can help establish a reliable molecular chemo-taxonomic categorization of the reviewed genotypes. Our multidimensional approach resulted in separating the six genotypes into two main clusters depending on the HCA dendrogram: the first cluster (I) was divided into two sub-clusters. The first sub-cluster was split into two groups: the first group included *D. stramonium* form *stramonium* and form *tatula*, and the second group only included *D. stramonium* form *inermis*. The second sub-cluster had just *D. metel*, a monotypic variation, whereas the second cluster (II) featured the remaining two genotypes, *D. innoxia* and *D. ferox*. Other research on the biosystematics of the genus *Capparis* (Ewas, 2023) and DNA barcoding based on the analysis of the *matK* and *rbcL* genes (Mohamed, 2016; Pegiou et al., 2023), support these results. The strong evolutionary relationship among these three forms, even though *D. stramonium* form *inermis* is relatively distant from the other two forms, *stramonium* and *tatula*, supports the conclusions reached by Bye and Sosa (2013) (Bye and Sosa, 2013) who used DNA sequences analysis in conjunction with morphological description to classify the genus *Datura* at the molecular level. Similar results were found by Kamel et al. (2009), Elzayat et al. (2020) and Ewas (2023) (Kamel et al., 2009; Elzayat et al., 2020; Ewas, 2023). On the other hand, according to PC1, the three forms of *D. stramonium* (*stramonium*, *tatula*, and *inermis*) were classed as “outliers” and seen on the positive side, whereas *D. ferox* and *D. innoxia* were present on the negative side. This conclusion may be drawn based on the total phenolic, alkaloids, and individual contents, since the genotypes of each group had nearly same levels. These findings are in line with PCA and HCA results of *Capparis* species (Ewas, 2023).

This conclusion was supported by the results of total phenolic TPC, flavonoid TFC and alkaloid contents TAC, which indicated that *D. stramonium* form *tatula* had the highest TPA and TFA values, followed by the other two forms *stramonium* and *inermis* of the species *D. stramonium* and the lowest values of TAC. Conversely, both *D. ferox* and *D. innoxia* had the lowest values of TPC and TFC and the highest values of TAC. The harmonization of molecular and phytochemical markers within some of the studied *Datura* genotypes required further thorough chemical characterization. According to Sulaiman and Balachandran (2012) and Ewas, (2023) (Sulaiman and Balachandran, 2012; Ewas, 2023), flavonoids, a significant proportion of naturally existing phenolic compounds, may be observed in a variety of plant organs in either their unbound form or as glycosides. Flavonoids are crucial for plant protection, floral colors, staying alive, and skeletal support, as claimed by Deng and Lu (2017), Ewas et al. (2022) and Ewas, (2023) (Deng and Lu, 2017; Ewas et al., 2022; Ewas, 2023) Further effects of flavonoids such as anti-inflammatory, anti-osteoporosis, and cancer-preventing effects were further demonstrated for flavonoids (García-Pérez et al., 2018; García-Pérez et al., 2019; Ewas et al., 2022). Additionally, they have several health advantages, namely, cardio-protective, antiviral, antioxidant, and neuroprotective properties (Ullah et al., 2020). Five distinct flavonoid classes have been identified so far from several *Datura* species, indicating the abundance of flavonoids in this genus (Ibrahim et al., 2018). The present investigation’s results showed that the leaves of *D. innoxia* and *D. ferox* had lowest levels of total flavonoids. On the other hand, *D. stramonium* form *tatula*, and *stramonium* leaves had highest values of total flavonoid contents. These findings may be supported by the fact that changes in the quantity of phenolic compounds are associated to changes in the synthesis of flavonoid compounds, which are derivatives of phenolic compounds (Ewas et al., 2022; Ewas, 2023) Furthermore, the increased values of both total phenolic and flavonoid contents resulted in the purplish color of *D. stramonium* form *tatula*. This result was observed and indicated previously by Hassan and Amer (2019) (Hassan et al., 2019).

The TAs, including hyoscyamine and hyoscine, serve as significant medications derived from *Solanaceae* family (Jaremicz et al., 2013). These TAs have a chemotaxonomical value for the *Solanaceae*, especially *Datura* genotypes (Griffin and Lin, 2000). The results of Qualitative analysis via HPTLC revealed the distribution patterns of six tropine alkaloid compounds including scopolamine, scopoletin, atropine, hyoscyamine, cuscohygrine and anisodamine within the examined genotypes. The harmony of TA compounds in the three species (*stramonium, tatula*, and *inermis*) demonstrates that they are all fit in the same species. A slight exception to this conclusion is the lack of scopoletin in the form *inermis*. Based on this finding, in conjunction with the other morphological, genetic, and chemical taxonomic findings, form *inermis* should be modified from variety level to subspecies level, becoming *D. stramonium* subsp. *inermis*. Several prior investigations support our findings regarding the variance of TAs distribution patterns across *Datura* species (Berkov et al., 2006; Jaremicz et al., 2013; El-Said et al., 2016). For further investigation of TA spectrum in the roots of six *Datura* genotypes, GC-MS was caried out to examine the extracts of all genotypes. The results of GC-MS reported thirty-one TAs. The common TAs between the three *D. stramonium* forms *stramonium*, *tatula* and *inermis*, indicating a chemical similarity that is compatible with genetic relationships and belongs to the same species. An extra bonus are the seven TAs (7β-acetoxy-6β-benzoyloxy-3α-hydroxytropane, 6β,7β-dibenzoyloxy-3α-hydroxytropane, 6β,7β-dihydroxy-3α-(phenylacetoxy) tropane, 3α-benzoyloxy-6β,7β-dihydroxytropane, 6β-benzoyloxy-3α-(4-hydroxy-3,5-dimetoxybenzoyloxy) tropane, Acetylcholine and Muscarine), which were identified for the first time in the genus *Datura*. In contrast to the other *Datura* genotypes studied, these novel compounds are unique to both *D. innoxia* and *D. ferox*. At the chemical level, our findings are compatible with previous research, which has shown the diverse existence of a variety of TAs in *Datura* species (Berkov et al., 2006; Jaremicz et al., 2013). Furthermore, the sub-species *inermis* and the other two varieties, *stramonium* and *tatula*, are quite comparable in terms of the identified TA types. However, the content of these compounds in the species *inermis* is comparatively lower than those of the other two species. This finding supports the suggestion of reclassifying and rearranging the *inermis* variety to bring it up to the level of subsp. *inermis*. It is worth noting that many previous studies have confirmed the confusion in the classification of species belonging to the genus *Datura*. Globally, Dupin et al. (2017) (Dupin et al., 2017b) reported that the genus *Datura* includes 12 species, while Jiao et al. (2002) (Jiao et al., 2002) reported that it includes 14 species. Since Linnaeus (1753) (Linnaeus, 1753), there has been ongoing discussion on the taxonomic status of *D. stramonium*. In the initially published edition of Species Plantarum, he referred to *D. stramonium* by the Latin word “*Tatula*.” Nevertheless, *D. tatula* Linnaeus (1762: 256) was distinguished from *D. stramonium* in the subsequently published edition of Species Plantarum. Although some authors stuck to this approach, others like Haegi (1976) (Haegi, 1976) categorized *D. tatula* to be variety or forma within *D. stramonium*. The Egyptian Flora initially only included three *D. stramonium* varieties. In 1974, Täckholm described two varieties of the white-flowered *D. stramonium*: *D. stramonium* var. *stramonium* L., which had spiny capsules, and *D. stramonium* var. *inermis* Safford (1921: 176), which had smooth capsules. The violet-flowered form, *D. tatula*, was considered by her as a distinct species, with two varieties: *D. tatula* var. *tatula* with smooth capsules and *D. tatula* var. *inermis* Timmerman (1927: 574) with spiny capsules. Later, without considering the color of the petals or stem, Hepper (1998) and Boulos (2002) regarded *D. tatula* as a synonym of *D. stramonium* based on the capsule form and leaf indumentum. There are no infraspecific features in either classifications. Despite this species’ medical significance, its taxonomic identification was unresolved (Hassan et al., 2019). In accordance with Stuessy’s (1990) (Stuessy, 1990) classifications of infraspecific taxa, we would suggest to reclassify the variety *inermis* to the level subspecies of the same species as follows: *Datura stramonium* form *stramonium*; *Datura stramonium* form *tatula*; *Datura stramonium* subsp. *Inermis*. It is evident from the foregoing, how TAs may represent a valuable tool in terms of chemotaxonomy for the *Solanaceae*, particularly the genus *Datura* (Griffin and Lin, 2000; Berkov et al., 2006; Jaremicz et al., 2013; Serag et al., 2019).

The present study advances our knowledge regarding the relationship between TAs and the transcription level of its biosynthetic genes. At the molecular level, higher expression of the biosynthetic genes *H6H* and *TR2* in *D. stramonium* form *stramonium*, *tatula*, and *inermis* resulted in an increase in the synthesis of various TAs. In contrast, increased transcripts of *PMT*, *TR1*, and *AT4* in both *D. ferox* and *D. innoxia* were associated with increased accumulation levels of TAs, particularly those novel compounds identified for the first time in this study including (7β-acetoxy-6β-benzoyloxy-3α-hydroxytropane, 6β,7β-dibenzoyloxy-3α-hydroxytropane, 6β,7β-dihydroxy-3α-(phenylacetoxy) tropane, 3α-benzoyloxy-6β,7β-dihydroxytropane, 6β-benzoyloxy-3α-(4-hydroxy-3,5-dimetoxybenzoyloxy) tropane, Acetylcholine and Muscarine), when compared to the other genotypes examined. The correlation between the levels of TAs and the expression of their biosynthetic genes in all genotype under investigation was evident from the transcripts and synthesis analysis results. The initial step in the biosynthesis of scopolamine, the conversion of putrescine to N-methyl putrescine, is catalyzed by *PMT* (Rasi et al., 2024). In the production of scopolamine and hyoscyamine, *TR1* changes tropinone into tropine, an intermediate. *TR2* participates in the pseudo-tropine biosynthesis secondary pathway. The last stage in the biosynthesis of scopolamine, L-scopolamine, is produced by the catalysis of hyoscyamine and L-6-hydroxy hyoscyamine by *h6h* (Rasi et al., 2024). However, in terms of TA transcripts and synthesis, the species *D. metel* showed values that were in the middle of all the investigated *datura* genotypes. As can be seen from the foregoing, the variable accumulation of TAs within the *Datura* genotype clearly resulted from the wide variance in gene expression of TA biosynthetic genes. According to Rasi et al. (2024) (Rasi et al., 2024) induction of *AT4, ODC*, *PMT, MPO*, *UGT1*, *TR1, TR2, PYKS*, *CYP82M3*, *H6H* and *HDH* can enhance the synthesis of TAs, such as hyoscyamine and scopolamine, in certain species; however, this is not always the case for all Solanaceous plants (Singh et al., 2011; Hedayati et al., 2020; Zhang et al., 2022). Its significance in TA biosynthesis is further highlighted by the documented function of biosynthetic genes expression in increasing the amounts of tropine and tropinone, the initial forms of hyoscyamine and scopolamine. Notwithstanding *AT4, ODC*, *PMT, MPO*, *UGT1*, *TR1, TR2, PYKS*, *CYP82M3*, *H6H* and *HDH* overexpression in certain instances, the absence of improvement in hyoscyamine and scopolamine levels suggests that additional variables, including plant species, gene families, transcription levels, therapies, and ecological circumstances, are important determinants of the biosynthetic results (Rasi et al., 2024). Furthermore, the novel TAs were discovered for the first time in the genus *Datura*, and it is possible that the enhanced expression of TA biosynthetic genes is directly responsible for the accumulation of these compounds in both *D. innoxia* and *D. ferox*. GC/MS is a helpful and accurate technology for quickly separating and identifying complicated combinations of TAs (Haegi, 1976; Witte et al., 1987). The integration between biosynthetic genes expression and TAs accumulation has a molecular and chemotaxonomical value for the *Solanaceae*, especially *Datura* species (El Bazaoui et al., 2011).

## 5 Conclusion

The genus *Datura* is recognized as a substantial source of several secondary metabolite sorts with a wide range of therapeutic benefits. *Datura*, on the other hand, has a history of frequent taxonomic modifications. To enhance the medicinal usage of this genus, a reliable evaluation method of genotypes diversity and proximity must be developed that includes morphological, molecular, and chemical taxonomy. The taxonomic status rearrangement of the Egyptian *Datura* as well as the identification of novel TAs in the current study identified the phylogenetic relationships among our taxa. Based on our morphological, molecular, and chemical results, we found that the six examined Egyptian *Datura* genotypes had a similarity of 45%-79%. While the three forms of *D. stramonium* in Egypt (*stramonium*, *tatula* and *inermis*) are closely related taxa exhibiting significant similarity (up to 79, 57, and 60%), they show some marked differences up to (21, 43, and 40% dissimilarity), respectively. In accordance with Stuessy’s (1990) (Stuessy, 1990) classifications of infraspecific taxa, we would suggest to reclassify the variety *inermis* to the level subspecies of the same species as follows: *Datura stramonium* form *stramonium*; *Datura stramonium* form *tatula*; *Datura stramonium* subsp. *Inermis.* Additionally, the results of Gas chromatography mass spectrometry reported thirty-one TAs. Out of these thirty-one compounds, seven novel TAs were described for the first time in the genus *Datura*, which enhances the medical and economic value of these genotypes. Expression level of biosynthetic genes including *PMT*, *TR1*, *TR2*, *H6H*, *HDH* and *AT4* influenced on TAs accumulation within the examined genotypes. Since the six Egyptian genotypes’ seeds have been grown in the same location for this investigation, environmental variables have no effect on their phenoplasticity. This study is significant since drug development strategies and enhanced therapeutic usage in the genus *Datura* heavily depend on a comprehensive knowledge of the species and subspecies’ molecular and phytochemical variability. The origin of the six *Datura* genotypes that grow within Egyptian flora has to be clarified by more research.

## Data Availability

The original contributions presented in the study are included in the article/**Supplementary Material**. Further inquiries can be directed to the corresponding author.

## Glossary

CDDP: Conserved DNA-Derived Polymorphism
SCoT: Start Codon Targeted Polymorphism
ISSR: Inter simple sequence repeat
A1: Intrachromosomal Asymmetry
bi: average length of the short arms in each pair chromosome
Bi: average length of the long arms in each pair chromosome
A2: Intrachromosomal asymmetry
$\bar{\text{X}}$: average chromosomal length for every single sample
S: Standard deviation
DNA: Deoxyribonucleic Acid
RNA: Ribonucleic acid
PCR: Polymerase chain reaction
UV: Ultraviolet
GC-MS: Gas Chromatography Mass Spectro
HPTLC: High-performance thin-layer chromatography
PMT: Putrescine N-methyltransferase
TR1: tropinone reductase I
TR2: tropinone reductase II (), AT4 aromatic amino acid aminotransferase 4
HDH: hyoscyamine dehydrogenase
H6H: hyoscyamine 6 beta-hydroxylase
PCA: Principal Components Analysis
HCA: Hierarchical Cluster Analysis
