# Supplementary material for: A three-sided story: a biosystematic revision of genus Datura reveals novel tropane alkaloids for the first-time in certain species
Source: Front Plant Sci. 2025 May 2;16:1555237. doi: 10.3389/fpls.2025.1555237 (PMC12081466; doi:10.3389/fpls.2025.1555237)
Supplement: Supplementary file 1 [file DataSheet1.pdf]

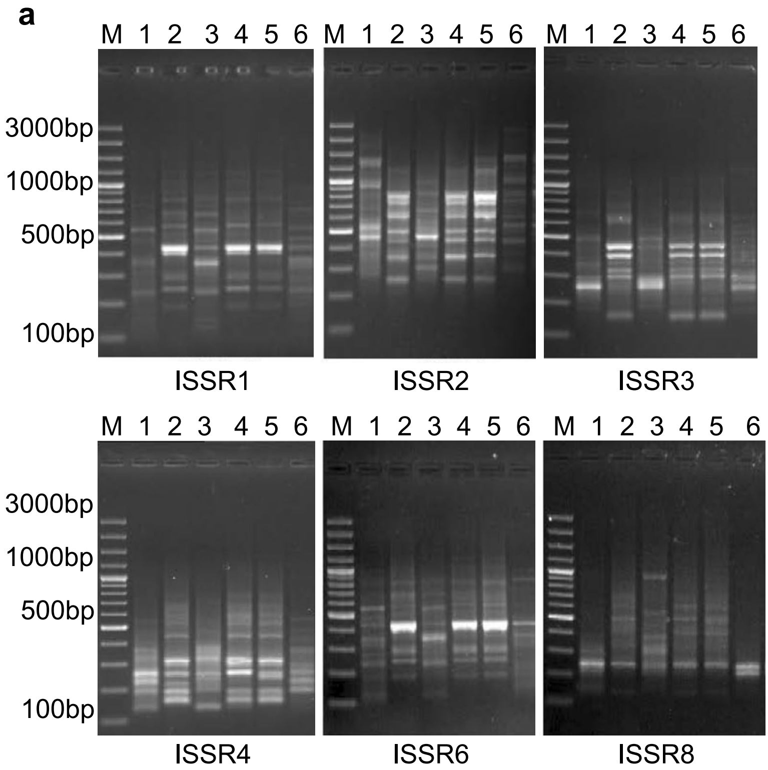
a
M 1 2 3 4 5 6
3000bp
1000bp
500bp
100bp
ISSR1
ISSR2
ISSR3
ISSR4
ISSR6
ISSR8

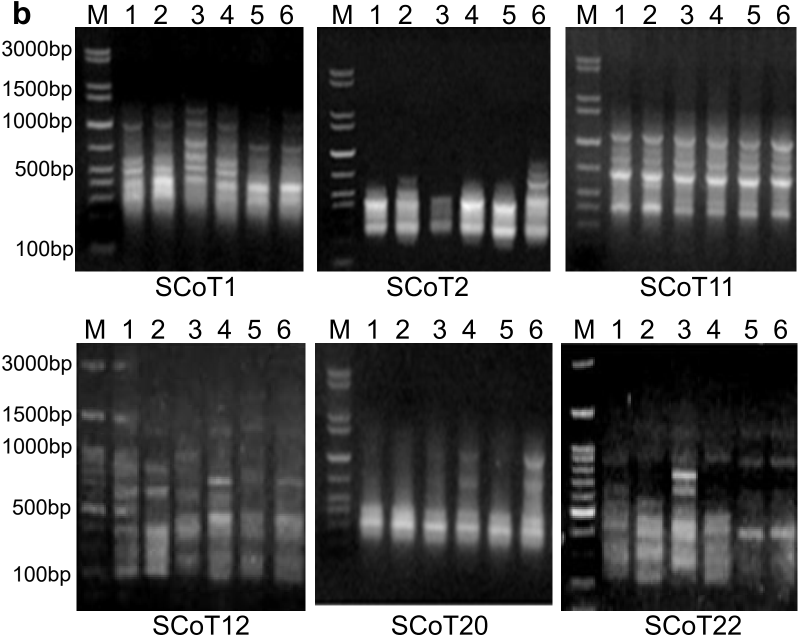

Supplementary Figure 1. PCR bands pattern of Egyptian genotypes; *D. stramonium* var. *stramonium* (1), var. *tatula* (2), subsp. *inermis* (3), *D. metel*, *D. ferox* and *D. innoxia* with **a.** ISSR primers: ISSR1, ISSR2, ISSR3, ISSR4, ISSR8 and ISSR8 along with **b.** SCoT primers: SCoT-1, SCoT-2, SCoT-11, SCoT-12, SCoT-20 and SCoT-22.

Supplementary table 1. Basic details and conservation status of the 6 *Datura* genotypes in this study.

Scientific name	Common name	Native occurrence	Status in Egypt	Habitat	IUCN status	References
Datura stramonium var. *stramonium*	Jimsonweed, Arotat	Mexico, United states of America, Jordan	Naturalised	most temperate and subtropical regions	unclassified[16]	wfo-0001021382
Datura stramonium var. *tatula*	Corn apple	Greek, tropical Mexico, America, India and Asia	Naturalised	most temperate and subtropical regions	unclassified	wfo-0001323479
Datura stramonium var. *inermis*	Thorn Apple, Jimsonweed, Jamestown Weed	Obscure	Introduced	Scrub jungles and wastelands and temperate regions	unclassified	wfo-0001021381
Datura innoxia	Downy thorn apple	Mexico	Introduced	Tropical and subtropical	Secure	wfo-0001021314
Datura metel	Gawz, Al-datura, Horn of plenty	Egypt	Native	Tropical and subtropical	unclassified	wfo-0001021339
Datura ferox	Fierce Thorn Apple	China	New record to Sinai	tropics and subtropics	unclassified	wfo-0001021301

Supplementary table 2. Name and sequences of the selected primers used in ISSR analysis profile.

Primer name	Sequence
ISSR1	5′-AGA GAG AGA GAG AGA GYC -3′
ISSR2	5′-AGA GAG AGA GAG AGA GYG -3′
ISSR3	5′-ACA CAC ACA CAC ACA CYT -3′
ISSR4	5′-ACA CAC ACA CAC ACA CYG -3′
ISSR6	5′-CGC GAT AGA TAG ATA GAT A-3′
ISSR8	5′-AGA CAG ACA GAC AGA CGC -3′

Supplementary table 3. Name and sequences of the selected primers used in SCoT analysis profile.

Primer name	Sequence
SCoT1	5′-CAACAATGGCTACCACCA-3′
SCoT2	5′-CAACAATGGCTACCACCC-3′
SCoT11	5′-AAGCAATGGCTACCACCA -3′
SCoT12	5′-ACGACATGGCGACCAACG-3′
SCoT20	5′-ACCATGGCTACCACCGCG-3′
SCoT22	5′-AACCATGGCTACCACCAC -3′

Supplementary table 4. Conserved DNA sequence targets and CDDPs primer sequences and details

Gene	Gene function	Primer name	Sequence (5' to 3')	GC (%)	Annealing temperature
ABP1	Auxin binding protein	ABP1	acsccatccaccgc	72	51
		ABP1-3	cacgaggacctscagg	68	50
PR1-1B	(Unknown) Low molecular weight protein	ZTL	agttccagggtgatctgc	74	54
		GR521461	accaaagacgtttccag	75	54
WRKY	Transcription factor related developmental and physiological functions	WRKYGQ	gtggttgtgtcttgcc	74	54
			gtggttgtgtcttgcc	61	51
GAPDH	Glyceradehyde-3-phospate dehydrogenase	GR518033	tctgaccgttacgagagg	73	53
			cttgagcttaccctcaga	72	53
MYB	Involved in secondary metabolism, environmental stresses, cellular morphogenesis	MYB1	ggcaagggctgccgc	79	54
		MYB2	ggcaagggctgccgg	79	54
ERF	Transcription factor implicated in plant disease resistance pathway	ERF1	cactaccccggsctscg	76	56
		ERF2	gcsgagatccgsgaccc	76	56

Supplementary table 5. Specific primers used in qRT-PCR.

Gene ID.	Primer sequences (5´-3´)
H6H	FW: GAACGACGCTGTAATGAGGAG
	RV: GTCAACTTCCTCACTTCCACT
PMT	FW: GCTTCGTTATCCTACCGTTG
	RV: ACGAGGATCATTAAAGTTAGCC
TR1	FW: CCTTGTTACTGGTGGCTCTAA
	RV: CCAAATTTCAAGGCATTCGT
TR2	FW: AGGAGCAATGGATCAACTCA
	RV: TCGACCAGAGAAGTTGCAATA
HDH	FW: CCATAAGTTTGATCTCATGTATGC
	RV: CAAGGTCCTCACGAGCCT
AT4	FW: GTTCCAGCTGTGCTGGCA
	RV: AGCTGGTTACTGATCCTTAA

Supplementary table 6. Band diversity and polymorphism % utilizing six ISSR primers in six *Datura* genotypes.

No.	ISSR primers	Total no. of bands	Monomorphic bands	Polymorphic bands	Unique bands	polymorphism %
1	ISSR1	23	4	18	1	78.36
2	ISSR2	21	6	14	1	66.14
3	ISSR3	18	3	14	3	77.56
4	ISSR4	16	4	11	0	67.58
5	ISSR6	18	3	14	4	77.56
6	ISSR8	12	2	9	1	73.27
**Total**		108	22	80	10	440.47
**Average**				8		62.92

Supplementary table 7. Mapping of positive specific markers for the six *Datura* genotypes using 6 ISSR primers

ISSR primers	Molecular size (bp)	var. *stramonium*	var. *tatula*	var. *inermis*	D. *innoxia*	D. *metel*	D. *ferox*	Positive marker
ISSR1	150	-	-	+	-	-	-	var. *inermis*
ISSR2	1110	-	-	+	-	-	-	*D. ferox*
ISSR3	1240	-	-	-	-	+	-	*D. metel*
	820	-	+	-	-	-	-	var. *tatula*
	680	+	-	-	-	-	-	var. *stramonium*
ISSR6	1310	-	-	-	-	-	+	*D. ferox*
	1190	-	-	-	-	+	-	*D. metel*
	320	-	-	-	-	+	-	*D. ferox*
	180	-	-	-	-	+	-	D. *innoxia*
ISSR8	550	-	-	+	-	-	-	var. *inermis*
**Range**	1310-150							
**Total**		1	1	3	0	4	1	10 (Positive markers)

Supplementary table 8. Genetic similarity % in 6 *Datura* genotypes using 6 ISSR Primers

**Genetic similarity**	var. *stramonium*	var. *tatula*	var. *inermis*	*D. innoxia*	*D. metel*	*D. ferox*
var. *stramonium*	1.0					
var. *tatula*	0.874	1.0				
var. *inermis*	0.852	0.746	1.0			
D. innoxia	0.514	0.467	0.441	1.0		
D. metel	0.589	0.511	0.523	0.442	1.0	
D. ferox	0.403	0.387	0.365	0.414	0.367	1.0

Supplementary table 9. Band diversity and polymorphism % utilizing six SCoT primers in six *Datura* genotypes

**No.**	**SCoT primers**	**Total no. of bands**	**Monomorphic bands**	**Polymorphic bands**	**Unique bands**	**polymorphism %**	**Genetic similarity %**
1	SCoT1	8	2	6	-	75.00	35.00
2	SCoT2	9	3	6	2	66.66	33.34
3	SCoT11	11	4	7	3	63.63	36.63
4	SCoT12	10	4	6	-	60.00	40.00
5	SCoT20	7	1	6	-	85.71	14.29
6	SCoT22	8	3	5	1	62.50	37.50
**Total**		53	17	36	5`	351.625	196.76
**Average**		8.83	2.8	6	0.83	58.60	32.79

Supplementary table 10. Band diversity and polymorphism % utilizing six CCDP primers in six *Datura* genotypes

CCDP Marker	Total bands	Polymorphic bands	Percentage of polymorphism	Polymorphic Information Content
**ABP1-1**	11	8	72.72	0.45
**ABP1-3**	7	5	71.42	0.44
**PR1-1F**	12	9	75.00	0.46
**PR1-R**	11	7	58.33	0.39
**WRKYF1**	10	7	58.33	0.39
**WRKYR1**	8	6	50.00	0.36
**GAPDHF**	7	5	41.66	0.19
**GAPDHR**	8	6	50.00	0.18
**MYB1F**	6	4	33.33	0.13
**MYB1R**	10	9	75.00	0.46
**ERF1F**	9	7	58.33	0.44
**ERF1R**	6	4	33.33	0.13
**Total**	105	77	627.45	3.63
**Average**	8.75	6.41	52.29	0.30

Supplementary table 11. Tropane alkaloids observed in *Datura* genotypes

**Alkaloid compounds**	**M⁺ & base peak**	**MS Ref.**	**Genotypes reported**					
			D. stramonium* var. *stramonium	***D. stramonium* var. *tatula***	**D. stramonium var. *inermis***	***D. metel***	***D. innoxia***	***D. ferox***
3-Acetoxy-6-hydroxytropane (A)	198, 95	(A)	+	+	+	+	+	+
3-Tygloyloxytropane (B)	222, 125	(A, B)	+	+	+	+	+	+
3α-Phenylacetoxytropane (C)	258, 125	(B)	+	+	+	+	-	-
3β-Phenylacetoxytropane (D)	258, 125	(B)	+	+	+	+	-	-
3- Tygloyloxy-6,7-dihydroxytropane (E)	224, 95	(A, B)	+	+	+	-	-	+
3-Hydroxy-6-tygloyloxytropane (F)	238, 114	(A)	+	+	-	+	-	-
3-Tygloyloxy-6-hydroxytropane (G)	238, 95	(A)	+	-	-	-	-	+
3-Tygloyloxy-6-isobutyryloxytropane (H)	310, 95	(A)	+	+	+	+	-	-
3α-Apotropoyloxytropane (I)	270, 125	(A, B)	+	-	-	+	+	+
3β -Apotropoyloxytropane (J)	270, 125	(A, B)	+	-	+	-	+	-
3-Tygloyloxy-6-methylbutyryloxytropane (K)	322, 95	(A)	+	-	-	-	-	-
Alkaloid 1 (L)	-, 125	-	+	+	+	-	+	+
Alkaloid 325 (M)	325, 95	(D)	+	-	-	-	-	-
Alkaloid 2 (N)	-, 95	-	+	-	-	-	-	-
3-Apotropoyloxy-6,7-epoxytropane (O)	284, 95	(A)	+	+	+	-	-	+
3-Tropoyloxytropane (P)	290, 125	(A, B)	+	-	-	+	+	+
3,6-Ditygloyloxytropane (**Q**)	320, 95	(A, B)	+	+	+	-	-	-

3α-Tygloyloxy-6-isovaleroyloxy-7-hydroxytropane (R)	340, 95	(G, I)	+	+	-	+	-	-
3β-Tygloyloxy-6-isovaleroyloxy-7-hydroxytropane (**S**)	340, 95	(G, I)	+	+	+	-	+	-
Methylscopolamine (T)	316, 95	(B)	+	-	+	-	-	+
3-Tropoyloxy-6,7-epoxynortropane (V)	290, 121	(B)	+	+	-	+	-	-
3-Tropoyloxy-6,7-epoxytropane (W)	300, 95	(A, B)	+	-	+	-	+	-
3α,6β-Ditygloyloxy-7β-hydroxytropane (X)	335, 95	(A, B)	+	+	+	-	-	+
3-Tropoyloxy-6-tygloyloxytropane (Y)	390, 95	(A, B)	+	+	+	+	+	+
7β-acetoxy-6β-benzoyloxy-3α-hydroxytropane			-	-	-	-	+	+
6β,7β-dibenzoyloxy-3α-hydroxytropane			-	-	-	-	+	+
6β,7β-dihydroxy-3α-(phenylacetoxy)tropane			-	-	-	-	+	+
3α-benzoyloxy-6β,7β-dihydroxytropane			-	-	-	-	+	+
6β-benzoyloxy-3α-(4-hydroxy-3,5-dimetoxybenzoyloxy)tropane			-	-	-	-	+	+
Acetylcholine			-	-	-	-	+	+
Muscarine			-	-	-	-	+	+
