# Supplementary material for: A three-sided story: a biosystematic revision of genus Datura reveals novel tropane alkaloids for the first-time in certain species
Source: Front Plant Sci. 2025 May 2;16:1555237. doi: 10.3389/fpls.2025.1555237 (PMC12081466; doi:10.3389/fpls.2025.1555237)
Supplement: Supplementary file 2 [file DataSheet2.pdf]

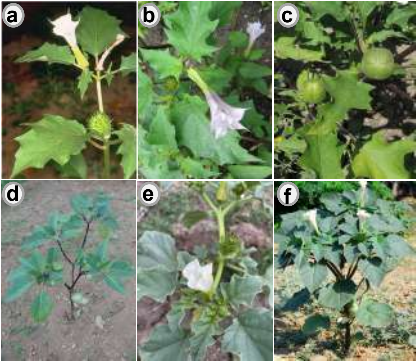

Supplementary Figure 1. Morphological characterisation for the leaves, stems, flowers, and capsules of Egyptian Datura genotypes including, **a.** *D. stramonium* form *stramonium;* **b.** *D. stramonium* form *tatula;* **c.** *D. stramonium* subsp. *Inermis*; **d.** *D. metel;* e. *D. ferox;* **f.** *D. innoxia*.

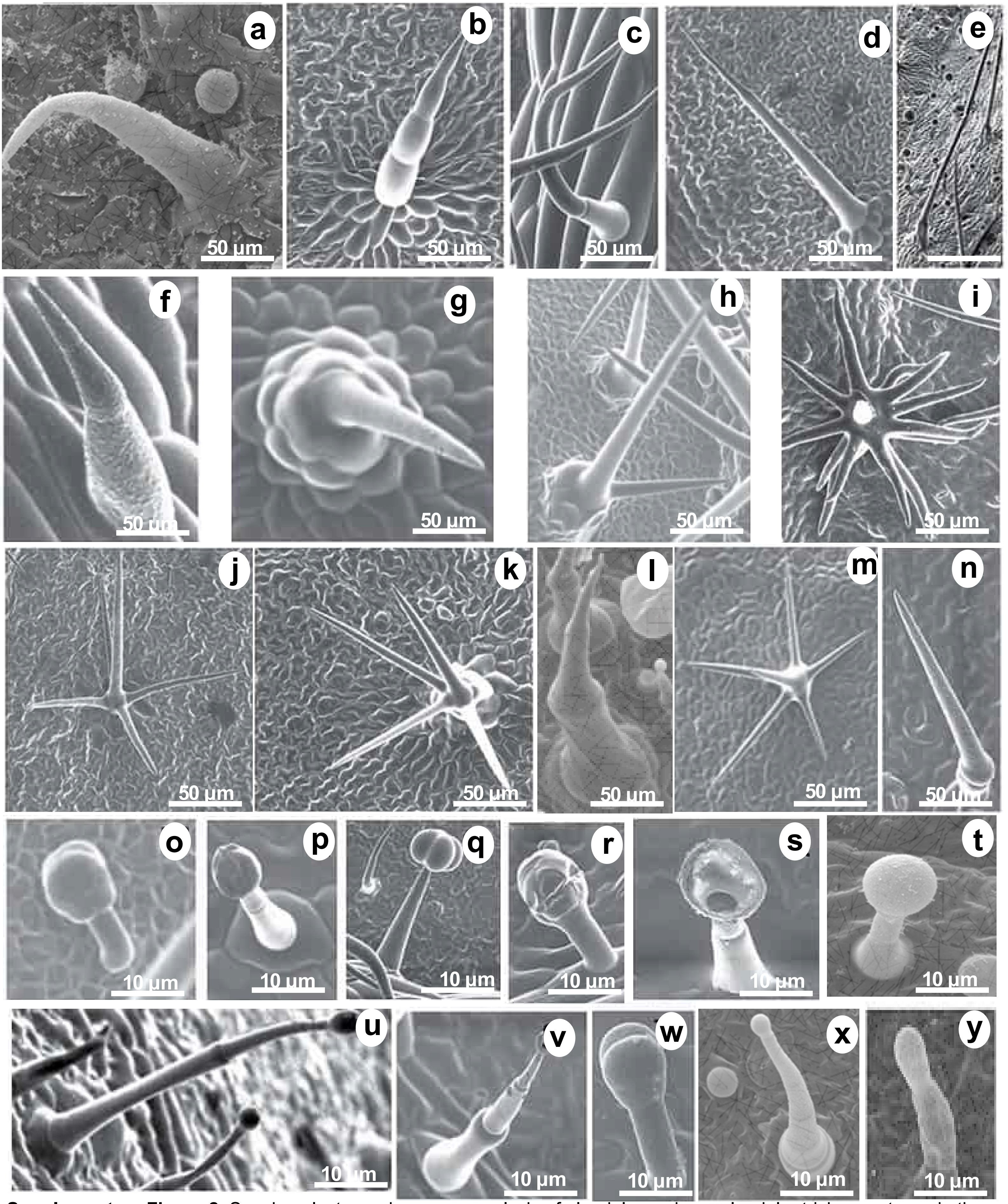

Supplementary Figure 2. Scaning electron microscopy analysis of glandular and non glandular trichomes types in the leaves of six studied Datura genotypes. The SEM indicates the presence of most glandular and eglandular trichomes types arranged from **a-y** including types I, II, III, IV, V, VI, III like b. scale bars = 50 µm (**a-n**), and = 10 µm (**o-y**).

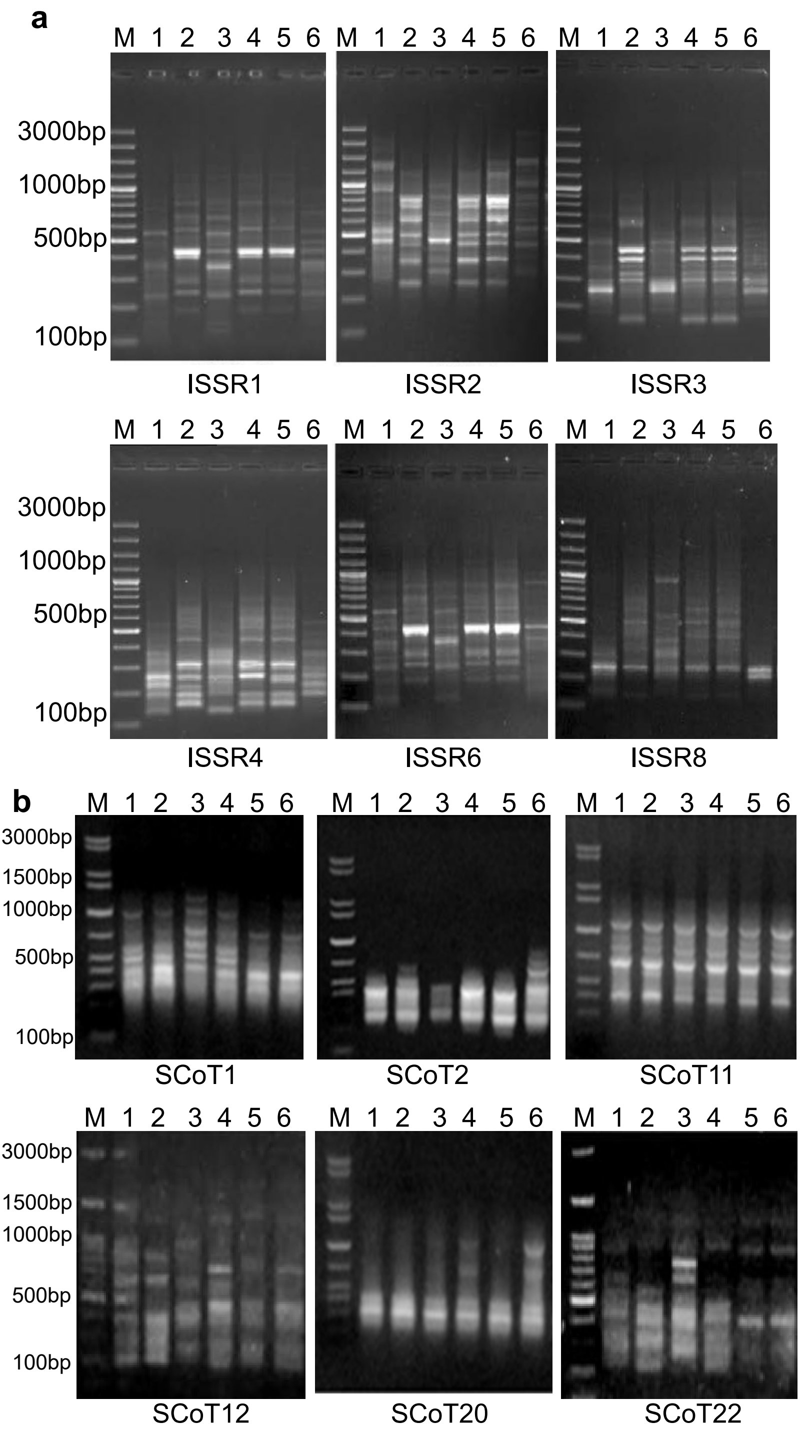

Supplementary Figure 3. PCR bands pattern of Egyptian genotypes; *D. stramonium* var. *stramonium* (1), var. *tatula* (2), subsp. *inermis* (3), *D. metel*, *D. ferox* and *D. innoxia* with **a.** ISSR primers: ISSR1, ISSR2, ISSR3, ISSR4, ISSR8 and ISSR8 along with **b.** SCoT primers: SCoT-1, SCoT-2, SCoT-11, SCoT-12, SCoT-20 and SCoT-22.

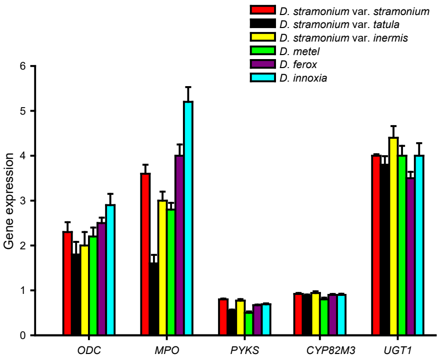

Supplementary fig. 4. Expression levels of tropane alkaloid biosynthesis genes in roots of Datura genotypes. *Ornithine decarboxylase (ODC), N-methylputrescine oxidase (MPO), type III polyketide synthase (PYKS), tropinone synthase (CYP82M3), and phenyllactate UDP-glycosyltransferase (UGT1)*. The data shown are the mean ± SE (n = 3).
