# Supplementary material for: A three-sided story: a biosystematic revision of genus Datura reveals novel tropane alkaloids for the first-time in certain species
Source: Front Plant Sci. 2025 May 2;16:1555237. doi: 10.3389/fpls.2025.1555237 (PMC12081466; doi:10.3389/fpls.2025.1555237)
Supplement: Supplementary file 3 [file DataSheet3.pdf]

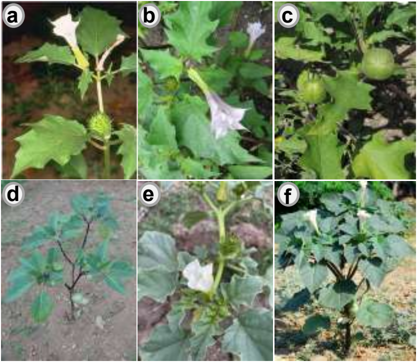

Supplementary Figure 1. Morphological characterisation for the leaves, stems, flowers, and capsules of Egyptian Datura genotypes including, **a.** *D. stramonium* form *stramonium;* **b.** *D. stramonium* form *tatula;* **c.** *D. stramonium* subsp. *Inermis*; **d.** *D. metel;* e. *D. ferox;* **f.** *D. innoxia*.

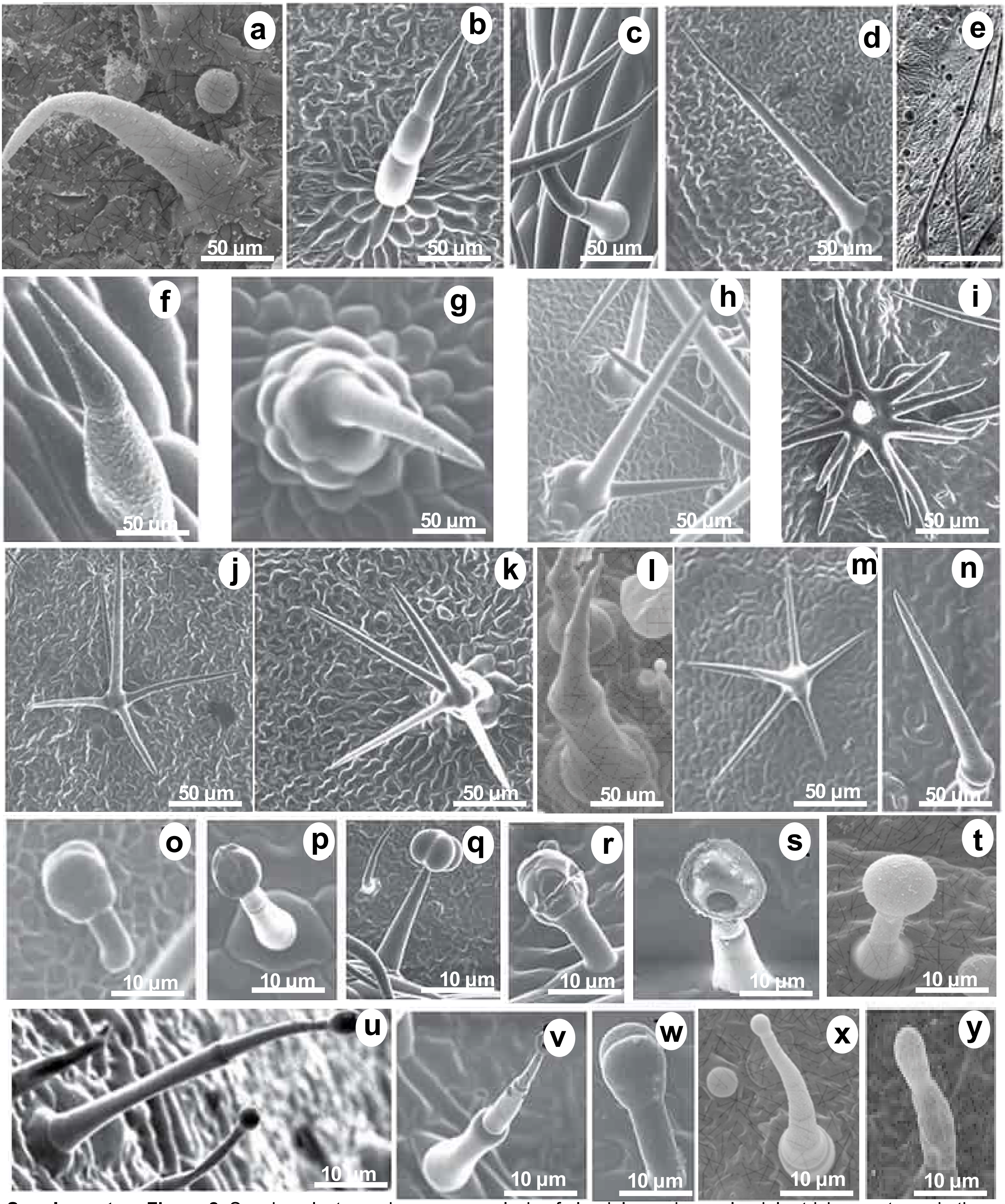

Supplementary Figure 2. Scaning electron microscopy analysis of glandular and non glandular trichomes types in the leaves of six studied Datura genotypes. The SEM indicates the presence of most glandular and eglandular trichomes types arranged from **a-y** including types I, II, III, IV, V, VI, III like b. scale bars = 50 µm (**a-n**), and = 10 µm (**o-y**).

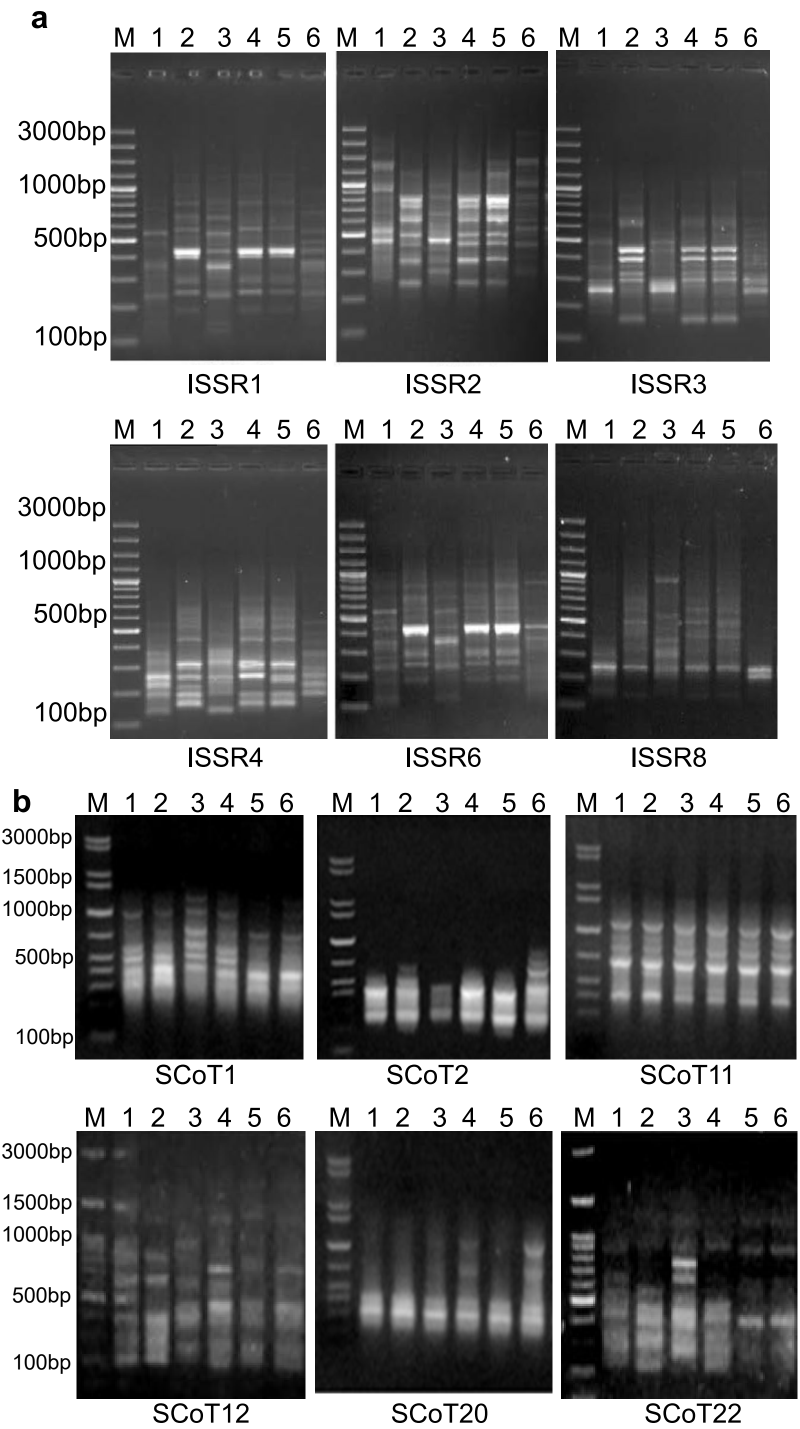

Supplementary Figure 3. PCR bands pattern of Egyptian genotypes; *D. stramonium* var. *stramonium* (1), var. *tatula* (2), subsp. *inermis* (3), *D. metel*, *D. ferox* and *D. innoxia* with **a.** ISSR primers: ISSR1, ISSR2, ISSR3, ISSR4, ISSR8 and ISSR8 along with **b.** SCoT primers: SCoT-1, SCoT-2, SCoT-11, SCoT-12, SCoT-20 and SCoT-22.

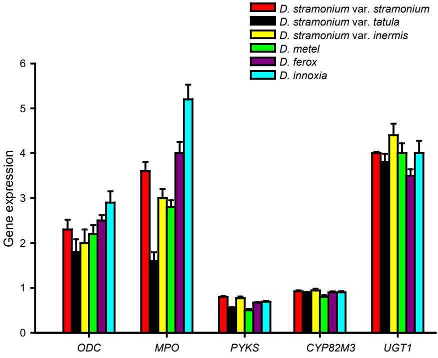

Supplementary fig. 4. Expression levels of tropane alkaloid biosynthesis genes in roots of Datura genotypes. *Ornithine decarboxylase (ODC), N-methylputrescine oxidase (MPO), type III polyketide synthase (PYKS), tropinone synthase (CYP82M3), and phenyllactate UDP-glycosyltransferase (UGT1)*. The data shown are the mean ± SE (n = 3).

Supplementary table 1. Basic details and conservation status of the 6 *Datura* genotypes in this study.

Scientific name	Common name	Native occurrence	Status in Egypt	Habitat	IUCN status	References
Datura stramonium var. *stramonium*	Jimsonweed, Arotat	Mexico, United states of America, Jordan	Naturalised	most temperate and subtropical regions	unclassified[16]	wfo-0001021382
Datura stramonium var. *tatula*	Corn apple	Greek, tropical Mexico, America, India and Asia	Naturalised	most temperate and subtropical regions	unclassified	wfo-0001323479
Datura stramonium var. *inermis*	Thorn Apple, Jimsonweed, Jamestown Weed	Obscure	Introduced	Scrub jungles and wastelands and temperate regions	unclassified	wfo-0001021381
Datura innoxia	Downy thorn apple	Mexico	Introduced	Tropical and subtropical	Secure	wfo-0001021314
Datura metel	Gawz, Al-datura, Horn of plenty	Egypt	Native	Tropical and subtropical	unclassified	wfo-0001021339
Datura ferox	Fierce Thorn Apple	China	New record to Sinai	tropics and subtropics	unclassified	wfo-0001021301

Supplementary table 2. Characteristic morphological features of the studied forms of Genus *Datura* (significant differential data are in *italics*).

Character	***D. stramonium* var. *stramonium***	***D. stramonium* var. *tatula***	***D. stramonium* subsp. *inermis***	***D. innoxia***	***D. metel***	***D. ferox***
Stem characters						
Hight	Up to 1m	Less than 1m	Less than 1m	0.5–1.5m	Up to 1.5m	Less than 1m
Habitat	erect	erect	Erect	Spreading	Spreading	Erect
Colour	Yellowish green	Purplish green	Yellowish green	Greyish green	Dark green to purplish	Yellowish green
Leaf characters						
Shape	Ovate-elliptic	Elliptic-ovate	Ovate-elliptic	Elliptic-ovate	Ovate-elliptic	Broadly ovate
Length	Up to 150 mm	Less than 150 mm	Up to 150 mm	100–200 mm	150–200 mm	100–200 mm
Width	Up to 120 mm	Less than 120 mm	Up to 120 mm	Up to 120 mm	Up to 150 mm	Up to 120 mm
Hairs on abaxial surface of leaf	Spare	Sparse	Absent	Sparse	Absent	Dense
Hairs on adaxial surface of leaf	Dense	Dense	Absent	Sparse	Absent	Dense
Trichome type on leaf blade	Eglandular & Glandular	Eglandular & Glandular	Absent	Eglandular	Eglandular	Eglandular
Flower characters						
Pedicel length	< 10 mm	≤ 10 mm	< 10 mm	10–20 mm	15–20 mm	10–15 mm
Calyx length	≤ 40 mm	≤ 50 mm	≤ 40 mm	50–70 mm	50–70 mm	30–50 mm
Calyx width	15 mm	≤ 15 mm	15 mm	15–20 mm	20–25 mm	10–15 mm
Calyx colour	light green	Purplish green	Light green	Yellowish green	Light green to purplish	Light green
Calyx teeth length	≤ 6 mm	≤ 8mm	≤ 6 mm	≤ 8 mm	≤ 10 mm	≤ 6 mm
Calyx teeth number	5 or 6	5	5	5 or 6	5 or 6	5
Corolla length	< 100 mm	≤ 100 mm	≤ 100 mm	100–150 mm	150–200 mm	100–150 mm
Corolla colour	White	Violet	White	White	White, sometimes purplish	White
Spine length	Up to 10 mm	Up to 9 mm	Up to 11 mm	Up to 12 mm	Up to 8 mm	< 8 mm
Spine numbers	> 50 / valve	≤ 50 / valve	≤ 50 / valve	> 50 / valve	< 50 / valve	< 50 / valve
Seed characters						
Seed length	3.5-5 mm	3.5-5 mm	3.5–5 mm	5–7 mm	5–7 mm	5–7 mm
Seed shape	reniform	reniform	Reniform	Reniform	Reniform	Reniform
Mature seeds colour	dark brown-black	dark brown-black	Dark brown-black	Black	Black	Black
Pollen grain characters						
Polar axis (P)	23.31-34.79 (29.07) µm	25.97-32.20 (28.98) µm	23.31–34.79 (29.07) µm	30.00–40.00 (35.00) µm	30.00–40.00 (35.00) µm	28.00–35.00 µm
Shape	prolate-spheroidal	prolate-spheroidal	Prolate-spheroidal	Prolate-spheroidal	Prolate-spheroidal	Prolate-spheroidal

Supplementary table 3. Name and sequences of the selected primers used in ISSR analysis profile.

Primer name	Sequence
ISSR1	5′-AGA GAG AGA GAG AGA GYC -3′
ISSR2	5′-AGA GAG AGA GAG AGA GYG -3′
ISSR3	5′-ACA CAC ACA CAC ACA CYT -3′
ISSR4	5′-ACA CAC ACA CAC ACA CYG -3′
ISSR6	5′-CGC GAT AGA TAG ATA GAT A-3′
ISSR8	5′-AGA CAG ACA GAC AGA CGC -3′

Supplementary table 4. Name and sequences of the selected primers used in SCoT analysis profile.

Primer name	Sequence
SCoT1	5′-CAACAATGGCTACCACCA-3′
SCoT2	5′-CAACAATGGCTACCACCC-3′
SCoT11	5′-AAGCAATGGCTACCACCA -3′
SCoT12	5′-ACGACATGGCGACCAACG-3′
SCoT20	5′-ACCATGGCTACCACCGCG-3′
SCoT22	5′-AACCATGGCTACCACCAC -3′

Supplementary table 5. Conserved DNA sequence targets and CDDPs primer sequences and details

Gene	Gene function	Primer name	Sequence (5’ to 3’)	GC (%)	Annealing temperature
ABP1	Auxin binding protein	ABP1	acsccatccaccgc	72	51
		ABP1-3	cacgaggacctscagg	68	50
PR1-1B	(Unknown) Low molecular weight protein	ZTL	agttccagggtgatctgc	74	54
		GR521461	accaaagacgtttccag	75	54
WRKY	Transcription factor related developmental and physiological functions	WRKYGQ	gtggttgtgtcttgcc	74	54
			gtggttgtgtcttgcc	61	51
GAPDH	Glyceradehyde-3-phospate dehydrogenase	GR518033	tctgaccgttacgagagg	73	53
			cttgagcttaccctcaga	72	53
MYB	Involved in secondary metabolism, environmental stresses, cellular morphogenesis	MYB1	ggcaagggctgccgc	79	54
		MYB2	ggcaagggctgccgg	79	54
ERF	Transcription factor implicated in plant disease resistance pathway	ERF1	cactaccccggsctscg	76	56
		ERF2	gcsgagatccgsgaccc	76	56

Supplementary table 6. Specific primers used in qRT-PCR.

Gene ID.	Primer sequences (5´-3´)
H6H	FW: GAACGACGCTGTAATGAGGAG
	RV: GTCAACTTCCTCACTTCCACT
PMT	FW: GCTTCGTTATCCTACCGTTG
	RV: ACGAGGATCATTAAAGTTAGCC
TR1	FW: CCTTGTTACTGGTGGCTCTAA
	RV: CCAAATTTCAAGGCATTCGT
TR2	FW: AGGAGCAATGGATCAACTCA
	RV: TCGACCAGAGAAGTTGCAATA
HDH	FW: CCATAAGTTTGATCTCATGTATGC
	RV: CAAGGTCCTCACGAGCCT
AT4	FW: GTTCCAGCTGTGCTGGCA
	RV: AGCTGGTTACTGATCCTTAA

Supplementary table 7. Band diversity and polymorphism % utilizing six ISSR primers in six *Datura* genotypes.

No.	ISSR primers	Total no. of bands	Monomorphic bands	Polymorphic bands	Unique bands	polymorphism %
1	ISSR1	23	4	18	1	78.36

2	ISSR2	21	6	14	1	66.14
3	ISSR3	18	3	14	3	77.56
4	ISSR4	16	4	11	0	67.58
5	ISSR6	18	3	14	4	77.56
6	ISSR8	12	2	9	1	73.27
**Total**		108	22	80	10	440.47
**Average**				8		62.92

Supplementary table 8. Mapping of positive specific markers for the six *Datura* genotypes using 6 ISSR primers

**ISSR primers**	**Molecular size (bp)**	**var. *stramonium***	**var. *tatula***	**var. *inermis***	**D. *innoxia***	**D. *metel***	**D. *ferox***	**Positive marker**
ISSR1	150	-	-	+	-	-	-	var. *inermis*
ISSR2	1110	-	-	+	-	-	-	*D. ferox*
ISSR3	1240	-	-	-	-	+	-	*D. metel*
	820	-	+	-	-	-	-	var. *tatula*
	680	+	-	-	-	-	-	var. *stramonium*
ISSR6	1310	-	-	-	-	-	+	*D. ferox*
	1190	-	-	-	-	+	-	*D. metel*
	320	-	-	-	-	+	-	*D. ferox*
	180	-	-	-	-	+	-	D. *innoxia*
ISSR8	550	-	-	+	-	-	-	var. *inermis*
**Range**	1310-150							
**Total**		1	1	3	0	4	1	10 (Positive markers)

Supplementary table 9. Genetic similarity % in 6 *Datura* genotypes using 6 ISSR Primers

**Genetic similarity**	var. *stramonium*	var. *tatula*	var. *inermis*	*D. innoxia*	*D. metel*	*D. ferox*
var. *stramonium*	1.0					
var. *tatula*	0.874	1.0				
var. *inermis*	0.852	0.746	1.0			
D. innoxia	0.514	0.467	0.441	1.0		
D. metel	0.589	0.511	0.523	0.442	1.0	
D. ferox	0.403	0.387	0.365	0.414	0.367	1.0

Supplementary table 10. Band diversity and polymorphism % utilizing six SCoT primers in six *Datura* genotypes

**No.**	**SCoT primers**	**Total no. of bands**	**Monomorphic bands**	**Polymorphic bands**	**Unique bands**	**polymorphism %**	**Genetic similarity %**
1	SCoT1	8	2	6	-	75.00	35.00
2	SCoT2	9	3	6	2	66.66	33.34
3	SCoT11	11	4	7	3	63.63	36.63
4	SCoT12	10	4	6	-	60.00	40.00
5	SCoT20	7	1	6	-	85.71	14.29
6	SCoT22	8	3	5	1	62.50	37.50
**Total**		53	17	36	5`	351.625	196.76
**Average**		8.83	2.8	6	0.83	58.60	32.79

Supplementary table 11. Band diversity and polymorphism % utilizing six CCDP primers in six *Datura* genotypes

**CCDP Marker**	**Total bands**	**Polymorphic bands**	**Percentage of polymorphism**	**Polymorphic Information Content**
**ABP1-1**	11	8	72.72	0.45
**ABP1-3**	7	5	71.42	0.44
**PR1-1F**	12	9	75.00	0.46
**PR1-R**	11	7	58.33	0.39
**WRKYF1**	10	7	58.33	0.39
**WRKYR1**	8	6	50.00	0.36
**GAPDHF**	7	5	41.66	0.19
**GAPDHR**	8	6	50.00	0.18

**MYB1F**	6	4	33.33	0.13
**MYB1R**	10	9	75.00	0.46
**ERF1F**	9	7	58.33	0.44
**ERF1R**	6	4	33.33	0.13
**Total**	105	77	627.45	3.63
**Average**	8.75	6.41	52.29	0.30

Supplementary table 12. Tropane alkaloids observed in *Datura* genotypes

**Alkaloid compounds**	**M+ & base peak**	**Characteristic ions (abundance %)**	**MS Ref.**	**Genotypes**					
				D. stramonium* var. *stramonium	***D. stramonium* var. *tatula***	***D. stramonium* var. *inermis***	***D. metel***	***D. ferox***	***D. innoxia***
3-Acetoxy-6-hydroxytropane (A)	198, 95	155(9), 140(22), 122(5), 95(59), 94(100), 82(8), 42(39)	(A)	+	+	+	+	+	+
3-Tygloyloxytropane (B)	222, 125	208(7), 140(9), 124(100), 96(18), 94(34), 83(51), 42(40)	(A, B)	+	+	+	+	+	+
3α-Phenylacetoxytropane (C)	258, 125	140(11), 124(100), 94(20) 91(18), 83(29), 82(32), 67(12)	(B)	+	+	+	+	-	-
3β-Phenylacetoxytropane (D)	258, 125	140(11), 124(100), 94(20) 91(18), 83(29), 82(32), 67(12)	(B)	+	+	+	+	-	-
3- Tygloyloxy-6,7-dihydroxytropane (E)	224, 95	223(0.2), 209(4), 140(12), 127(2), 98(10), 84(100), 55(4)	(A, B)	+	+	+	-	-	+
3-Hydroxy-6-tygloyloxytropane (F)	238, 114	195(3), 140(22), 95(81), 94(100), 83(7), 55(21), 42(30)	(A)	+	+	-	+	-	-
3-Tygloyloxy-6-hydroxytropane (G)	238, 95	195(3), 140(22), 95(81), 94(100), 83(7), 55(21), 42(30)	(A)	+	-	-	-	-	+
3-Tygloyloxy-6-isobutyryloxytropane (H)	310, 95	210(10), 209(5), 138(10), 122(40), 95(100),94(99), 55(23), 43(27)	(A)	+	+	+	+	-	-
3α-Apotropoyloxytropane (I)	270, 125	140(14), 124(100), 103(13), 95(66), 94(21), 83(49), 82(49), 42(26)	(A, B)	+	-	-	+	+	+

3β -Apotropoyloxytropane (J)	270, 125	140(14), 124(100), 103(13), 95(66), 94(21), 83(49), 82(49), 42(26)	(A, B)	+	-	+	-	+	-
3-Tygloyloxy-6-methylbutyryloxytropane (K)	322, 95	238(4), 222(8), 195(4), 138(15), 122(30), 94(100), 83(20), 55(30)	(A)	+	-	-	-	-	-
Alkaloid 1 (L)	-, 125		-	+	+	+	-	+	+
Alkaloid 325 (M)	325, 95	307(15), 237(6), 220(11), 138(65), 137(52), 94(100)	(D)	+	-	-	-	-	-
Alkaloid 2 (N)	-, 95		-	+	-	-	-	-	-
3-Apotropoyloxy-6,7-epoxytropane (O)	284, 95	154(41), 138(54), 136(29), 108(44), 103(44), 94(100), 81(21), 42(30)	(A)	+	+	+	-	-	+
3-Tropoyloxytropane (P)	290, 125	142(2), 140(5), 124(100), 103(5), 96(15), 94(16), 91(12), 83(12), 82(16), 42(7)	(A, B)	+	-	-	+	+	+
3,6-Ditygloyloxytropane (**Q**)	320, 95	238(4), 222(8), 195(4), 138(15), 122(30), 94(100), 83(20), 55(30)	(A, B)	+	+	+	-	-	-
3α-Tygloyloxy-6-isovaleroyloxy-7-hydroxytropane (R)	340, 95	240(3), 237(3), 222(2), 210(3), 154(6), 138(64), 137(40), 120(6), 94(100), 93(44), 85(4), 83(12), 55(16), 42(10)	(G, I)	+	+	-	+	-	-
3β-Tygloyloxy-6-isovaleroyloxy-7-hydroxytropane (**S**)	340, 95	240(3), 237(3), 222(2), 210(3), 154(6), 138(64), 137(40), 120(6), 94(100), 93(44), 85(4), 83(12), 55(16), 42(10)	(G, I)	+	+	+	-	+	-
Methylscopolamine (T)	316, 95	154(39), 138(81), 136(34), 108(34), 94(100), 81(19)	(B)	+	-	+	-	-	+
3-Tropoyloxy-6,7-epoxynortropane (V)	290, 121	142(2), 140(5), 124(100), 103(5), 96(15), 94(16), 91(12), 83(12), 82(16), 42(7)	(B)	+	+	-	+	-	-
3-Tropoyloxy-6,7-epoxytropane (W)	300, 95	272(4), 140(10), 124(100), 94(23), 82(21), 67(10)	(A, B)	+	-	+	-	+	-
3α,6β-Ditygloyloxy-7β-hydroxytropane (X)	335, 95	251(1), 236(4), 137(41), 137(55), 95(50), 94(100), 83(36), 55(42)	(A, B)	+	+	+	-	-	+
3-Tropoyloxy-6-tygloyloxytropane (Y)	390, 95	262(15), 223(39), 138(19), 122(47), 95(100), 94(98), 55(22)	(A, B)	+	+	+	+	+	+
7β-acetoxy-6β-benzoyloxy-3α-hydroxytropane (Z0)	288.369	261(14), 222(39), 138(19), 122(47), 95(100), 94(98), 55(22)	(A, B)	-	-	-	-	+	+

6β,7β-dibenzoyloxy-3α-hydroxytropane (Z1)	343.023	255(5), 182(43), 122(56), 103(12), 95(70), 94(100), 82(16), 55(24), 43(28)	(G)	-	-	-	-	+	+
6β,7β-dihydroxy-3α-(phenylacetoxy)tropane (Z2)	260.14	126(3), 124(30), 110(100), 105(1), 94(7), 82(17), 77(2)	(A)	-	-	-	-	+	+
3α-benzoyloxy-6β,7β-dihydroxytropane (Z3)	272	140(14), 124(100), 103(13), 95(66), 94(21), 83(49), 82(49), 42(26)	(B)	-	-	-	-	+	+
6β-benzoyloxy-3α-(4-hydroxy-3,5-dimetoxybenzoyloxy)tropane (Z4)	272	140(14), 124(100), 103(13), 95(66), 94(21), 83(49), 82(49), 42(26)	(B)	-	-	-	-	+	+
Acetylcholine (Z5)	146.207	102(6), 88(8), 84(100), 82(9), 70(8), 55(5), 42(28)	(J)	-	-	-	-	+	+
Muscarine (Z6)	174.26	133(15), 118(100), 94(23), 90(20), 81(62), 82(79), 67(20), 42(27)	(K)	-	-	-	-	+	+
